# Human adipose and umbilical cord mesenchymal stem cell-derived extracellular vesicles mitigate photoaging via TIMP1/Notch1

**DOI:** 10.1038/s41392-024-01993-z

**Published:** 2024-10-30

**Authors:** Huan Zhang, Xian Xiao, Liping Wang, Xianhao Shi, Nan Fu, Shihua Wang, Robert Chunhua Zhao

**Affiliations:** 1 Institute of Basic Medical Sciences Chinese Academy of Medical Sciences, School of Basic Medicine Peking Union Medical College, Beijing, China; 2 Department of Cell Biology, School of Life Sciences, Shanghai University, Shanghai, China

**Keywords:** Mesenchymal stem cells, Senescence

## Abstract

UVB radiation induces oxidative stress, DNA damage, and inflammation, leading to skin wrinkling, compromised barrier function, and an increased risk of carcinogenesis. Addressing or preventing photoaging may offer a promising therapeutic avenue for these conditions. Recent research indicated that mesenchymal stem cells (MSCs) exhibit significant therapeutic potential for various skin diseases. Given that extracellular vesicles (EV) can deliver diverse cargo to recipient cells and elicit similar therapeutic effects, we investigated the roles and underlying mechanisms of both adipose-derived MSC-derived EV (AMSC-EV) and umbilical cord-derived MSC-derived EV (HUMSC-EV) in photoaging. Our findings indicated that in vivo, treatment with AMSC-EV and HUMSC-EV resulted in improvements in wrinkles and skin hydration while also mitigating skin inflammation and thickness alterations in both the epidermis and dermis. Additionally, in vitro studies using human keratinocytes (HaCaTs), human dermal fibroblast cells (HDFs), and T-Skin models revealed that AMSC-EV and HUMSC-EV attenuated senescence, reduced levels of reactive oxygen species (ROS) and DNA damage, and alleviated inflammation induced by UVB. Furthermore, EV treatment enhanced cell viability and migration capacity in the epidermis and promoted extracellular matrix (ECM) remodeling in the dermis in photoaged cell models. Mechanistically, proteomics results showed that TIMP1 was highly expressed in both AMSC-EV and HUMSC-EV and could exert similar effects as MSC-EV. In addition, we found that EV and TIMP1 could inhibit Notch1 and downstream targets Hes1, P16, P21, and P53. Collectively, our data suggests that both AMSC-EV and HUMSC-EV attenuate skin photoaging through TIMP1/Notch1.

## Introduction

Human skin, the body’s largest organ, serves as the primary barrier against environmental factors, especially solar ultraviolet (UV) radiation.^1^ UV is divided into UVA (320–400 nm), UVB (280–20 nm), and UVC (280–100 nm) based on wavelength. UVC is absorbed by the stratospheric ozone layer and does not reach the earth. Relative to UVA, UVB has less penetrating ability into the skin but a greater damaging effect.^2^ UVB exposure increases the risk of various skin disorders leading to tanning, and inflammatory reactions including redness and blistering in the short term.^3^ In the long term, it can lead to photoaging including wrinkles, decreased barrier capacity and resilience, and an increased risk of carcinogenesis such as keratinocyte carcinoma, basal cell carcinoma, cutaneous squamous cell carcinoma, and melanoma.^4^ It is reported that nearly 80% of adults aged 70 and above suffer from one or more skin diseases that require further treatment or follow-up, including UV-induced skin diseases such as Lentigo senilis (69.5%), actinic keratosis (22.3%), Basal cell carcinoma (5.07%), Melanoma (0.54%) and Squamous cell carcinoma (0.36%).^5^ Therefore, an in-depth understanding of UVB-induced skin damage, molecular mechanisms, and therapeutic options is of great scientific importance and clinical value. Currently, there are several traditional treatments for photoaging, including the use of retinoids, antioxidant products, or optical therapies; however, some strategies are not always effective or can cause side effects, such as allergy or irritation.^6,7^ An effective treatment program with few side effects is therefore needed.

Extracellular vesicles (EV) are lipid bilayer membrane-delimited particles with functional components such as proteins and microRNAs (miRNAs).^8^ They exhibit diverse sizes, cargo compositions and surface markers, and are ubiquitously released from cells under both physiological and pathological conditions. In recent years, EV have received much scientific attention since they can mediate cell-to-cell communication and have shown great promise for the treatment of a number of diseases.^9^ For instance, in a first-in-human, double-blind, placebo-controlled, phase I clinical trial involving allogeneic, platelet-derived EV, it was evidence that these EV are safe and potentially applicable in patients with delayed or disrupted wound healing.^10^ In the realm of skin photoaging, researchers have embarked on investigations elucidating the significant roles of EV. Notably, Yi You et al. employed EVs generated through cellular nanoporation from human dermal fibroblasts to encapsulate COL1a1 mRNA and found these EV could induce the formation of collagen-protein grafts and reduce wrinkle formation in the collagen-depleted dermal tissue of mice with photoaged skin.^11^ In this study, we focus on EV derived from mesenchymal stem cells, which have protective activity against oxidative stress, cell apoptosis, and immunomodulatory properties.^12^ MSCs-derived EV (MSC-EV) have previously been documented to inherit similar therapeutic effects from MSCs. Compared to whole-cell therapy, EV possess numerous advantages, such as low immunogenicity, easy accessibility, effortless preservation, and absence of ethical concerns.^13^

MSCs exhibit remarkable healing capacities that apply to various skin diseases. MSCs contribute to various wound-healing processes and can migrate to the site of skin injury, inhibit inflammation, optimize collagen deposition, and increase the proliferation and differentiation potential of fibroblasts, epidermal cells, and endothelial cells.^14,15^ Application of MSCs for cutaneous burn treatment can promote burn wound healing and neovascularization.^16^ Moreover, several studies highlight the potential capability of MSCs in inhibiting the severity and development of psoriasis,^17–19^ mainly attributed to their anti-inflammatory and immunomodulatory properties.^20^ We have demonstrated that In the In vivo natural aging and type-2 diabetes mouse wound-healing models MSC-EV promoted wound closure and new blood vessel formation. Interestingly, in the natural aging mice wound-healing model, we noticed reduction of wrinkle formation in the skin around the wound.^21^ Given the immunomodulatory properties, ability to promote cell proliferation and differentiation, and regulation of extracellular matrix (ECM) dynamics reported in MSCs within the skin, combined with our observation of reduced wrinkle formation after MSC-EV application, we hypothesized that EV secreted by MSCs could also protect photoaging.

We aimed to investigate the effects of MSC-EV on both keratinocytes and fibroblasts in vitro, reconstructed-full-thickness skin model of photoaging, as well as photoaging nude mice in vivo. In addition, we investigated the underlying molecular mechanisms by EV proteomics and RNA-seq. Specifically, we pinpointed a key protein component-TIMP1 within MSC-EV cargo that mediated largely the anti-photoaging effects of MSC-EV. TIMP1 is a glycoprotein of the TIMP family, which includes four types of protease inhibitors known as TIMP1-4.^22^ It is reported that TIMP1 can directly inhibit the activity of matrix metalloproteinases (MMPs) and a disintegrin and metalloproteinases (ADAMs), thereby regulating ECM protein hydrolysis and matrix remodeling.^23^ Additionally, our findings indicate that the Notch signaling pathway exhibits significant alterations during skin photoaging, and treatments with EV influence these changes. Notch signaling is crucial for cell lineage selection, epidermal homeostasis, and skin function.^24^ The Notch signaling pathway in the skin promotes keratinocyte differentiation that culminates in skin barrier formation,^25^ while Notch in hair follicles balances their proliferation and differentiation.^26^ Our results suggest that MSC-EV can act as nanotherapeutic agents to rescue UVB-induced skin photoaging via the TIMP1/Notch1 pathway.

## Results

### Characterization of AMSC-EV and HUMSC-EV and their intense uptake in the skin

**Fig. 1:**
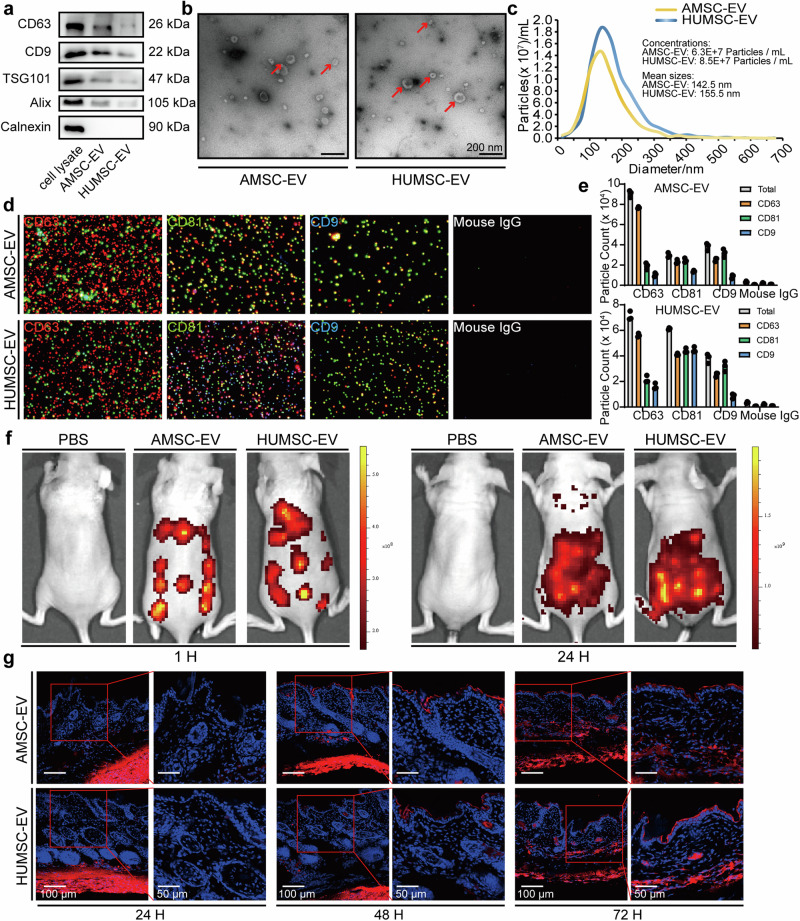
Characterization of AMSC-EV and HUMSC-EV and their intense uptake in the skin. **a** Western blot analysis for exosomal marker proteins CD63, CD9, TSG101, and Alix and cell-specific marker Calnexin. **b** Morphology of AMSC-EV and HUMSC-EV was examined by TEM (scale bar, 200 nm). **c** Size distribution profile of EV detected by NTA. **d** ExoView analysis detected CD63, CD81, and CD9. Mouse IgG was used as a negative control. **e** Quantitation of the expression of CD63, CD81, CD9, and Mouse IgG in EV. **f** Representative in vivo fluorescent imaging of PKH26-labeled EV in mice at 1 h and 24 h after injection. **g** Biodistribution of PKH26-labeled EV in mice skin at 24 h, 48 h and 72 h after EV treatment

MSCs could be isolated from a variety of tissues. In this study, we used two types of MSCs, human adipose-derived MSCs (hAMSCs) and umbilical cord-derived MSCs (hHUMSCs), and EV were isolated from the supernatants by ultracentrifugation. To characterize hAMSCs-derived EV (AMSC-EV) and hHUMSCs-derived EV (HUMSC-EV), we performed transmission electron microscopy (TEM), nanoparticle tracking analysis (NTA), western blot, and ExoView analysis. Both EV had enrichment of marker proteins, including CD63, CD9, TSG101, and Alix, and the absence of endoplasmic reticulum membrane marker calnexin (Fig. 1a). They presented a classic cup-shaped appearance under TEM (Fig. 1b). According to NTA, EV had a size distribution peaking at about 150 nm in diameter (Fig. 1c). Furthermore, ExoView analysis demonstrated high expression levels of EV transmembrane proteins CD63, CD81 and CD9 (Fig. 1d, e). No significant differences were observed between AMSC-EV and HUMSC-EV regarding morphology, size distribution, and marker expression patterns. We then investigated the distribution of EV in the skin by subcutaneous injection. Over time, in vivo imaging revealed the intense uptake of EV in the skin (Fig. 1f). Histological analysis of skin biopsies confirmed the dispersed presence of EV in the dermis (Fig. 1g). Additionally, no adverse events were detected in mice after MSC-EV injection as evidenced by histological examination of vital organs (Supplementary Fig. 1a), and blood biochemistry analysis of liver, renal and cardiac function indices (Supplementary Fig. 1b). Together, these results demonstrated a high sample purity of AMSC-EV and HUMSC-EV and showed EV intense uptake in the skin.

### AMSC-EV and HUMSC-EV mitigated photoaging of keratinocytes and fibroblasts in vitro

Exposure to UVB radiation instigates a cascade of skin aging processes, oxidative stress manifested as a dramatic increase in ROS levels,^27^ and extensive DNA damage that threatens genome stability and cell viability. To investigate whether AMSC-EV and HUMSC-EV have a protective effect on photoaging, we used human keratinocytes (HaCaTs), human primary keratinocytes (HKCs), and human dermal fibroblasts (HDFs) as in vitro cellular models.

**Fig. 2:**
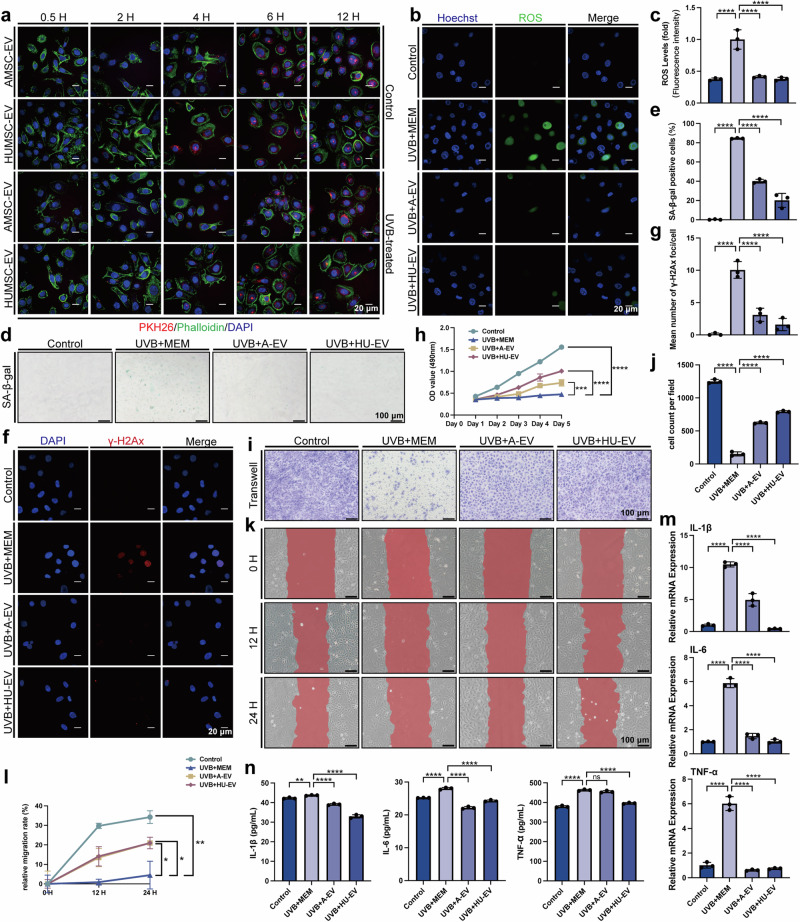
AMSC-EV and HUMSC-EV mitigated photoaging of HaCaTs in vitro. **a** Representative image of MSC-EV uptake by HaCaTs pretreated with or without UVB irradiation (scale bar, 20 μm). **b** Representative immunofluorescence staining images of positive cells of ROS (green) and DAPI (scale bar, 20 μm). **c** Fluorescence intensity of ROS levels. *n* = 3, ****P < 0.0001. **d** Representative images of SA-β-gal staining in HaCaTs (scale bar, 100 μm). **e** Quantitation of SA-β-gal positive cells in HaCaTs. **f** Representative immunofluorescence staining images of positive cells of γ-H2Ax (red) and DAPI (scale bar, 20 μm). **g** Quantitation of the mean number of γ-H2Ax foci/cell. *n* = 3, ****P < 0.0001. **h** Quantitation of HaCaTs proliferation detected by CCK8 assay with the OD value on Day 1, Day 2, Day 3, Day 4, and Day 5. *n* = 3, ***P < 0.001, ****P < 0.0001. **i** Representative images of transwell assays of HaCaTs (scale bar, 100 μm). **j** Quantitation of transwell assays of HaCaTs. *n* = 3, ****P < 0.0001. **k** Representative images of migration assay and the image was taken at the indicated times (scale bar, 100 μm). **l** Quantitation of migration assays of HaCaTs. *n* = 3, *P < 0.05, **P < 0.01. **m** Quantitation of IL-1β, IL-6, and TNF-α released by HaCaTs was detected through qRT-PCR. *n* = 3, ****P < 0.0001. **n** Quantitation of IL-1β, IL-6, and TNF-α released by HaCaTs was detected through ELISA. *n* = 3, **P < 0.01, ****P < 0.0001

HaCaTs are a spontaneously immortalized human keratinocyte line that can mimic the characteristics of normal epidermal cells, a major cell component in the epidermis, which is the outermost skin layer directly affected by UVB.^28^ We first investigated whether UVB irradiation affects the uptake of AMSC-EV and HUMSC-EV by HaCaTs. EV were labeled with PKH26 and the fluorescence intensity in both control and photoaged HaCaTs were compared. No significant differences were observed (Fig. 2a and Supplementary Fig. 2a), indicating that UVB irradiation did not affect the uptake of AMSC-EV and HUMSC-EV by HaCaTs. We then evaluated the effects of EV by analyzing various biomarkers related to photoaging. After irradiation, there was a notable increase in ROS levels, whereas treatment with EV reduced ROS levels (Fig. 2b, c). UVB exposure induced the accumulation of SA-β-gal positive cells in HaCaTs, which was attenuated by EV treatment (Fig. 2d, e), while senescence markers P16 and LMNB1 in HaCaTs were also rescued after EV stimulation (Supplementary Fig. 2b, c). The level of γ-H2Ax increased significantly after irradiation and notably decreased in the AMSC-EV and HUMSC-EV groups (Fig. 2f, g). Under physiological conditions, the proliferation and migration of epidermal cells play an essential role.^29^ CCK8 assay showed that proliferation was inhibited after irradiation, and AMSC-EV and HUMSC-EV had the effect of promoting proliferation (Fig. 2h). Both transwell and migration experiments showed that migration was affected by irradiation, and AMSC-EV and HUMSC-EV promoted migration (Fig. 2i–l). Additionally, there was a significant increase in inflammatory factors after UVB irradiation, significantly ameliorated by EV (Fig. 2m, n). Moreover, we utilized human primary keratinocytes (HKCs) to replicate key experiments involving senescence, yielding results consistent with those obtained from HaCaT cells (Supplementary Fig. 3a–f). The above results indicated that UVB can increase epidermal ROS, aging, DNA damage, proliferation, migration, and inflammatory response, while AMSC-EV and HUMSC-EV can alleviate these effects.

**Fig. 3:**
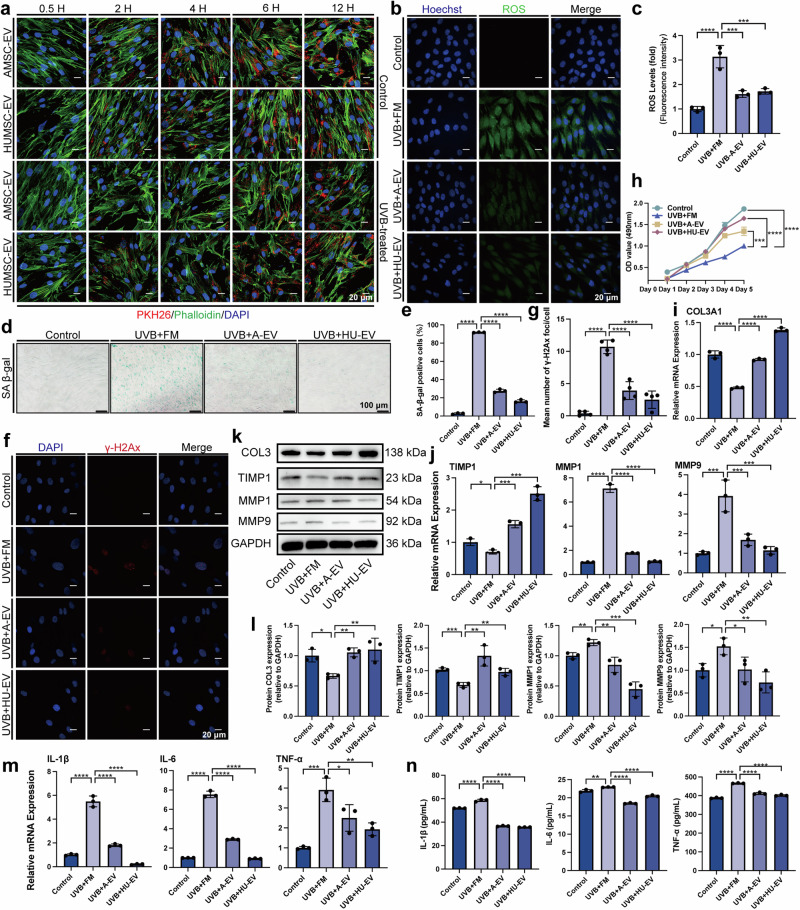
AMSC-EV and HUMSC-EV mitigated photoaging of HDFs in vitro. **a** Representative images of MSC-EV uptake by HDFs pretreated with or without UVB irradiation (scale bar, 20 μm). **b** Representative immunofluorescence staining images ROS (green) and DAPI (scale bar, 20 μm). **c** Fluorescence intensity of ROS levels. *n* = 3, ***P < 0.001, ****P < 0.0001. **d** Representative images of SA-β-gal staining in HDFs (scale bar, 100 μm). **e** Quantitation of SA-β-gal positive cells in HDFs. *n* = 3, ****P < 0.0001. **f** Representative immunofluorescence staining images of positive cells of γ-H2Ax (red) and DAPI (scale bar, 20 μm). **g** Quantitation of a mean number of γ-H2Ax foci/cell. **h** Quantitation of HDFs proliferation detected by CCK8 assay with the OD value on Day 1, Day 2, Day 3, Day 4, and Day 5. *n* = 3, ***P < 0.001, ****P < 0.0001. **i** Quantitative of COL31A in HDFs by qRT-PCR. *n* = 3, *P < 0.05, **P < 0.01. **j** Quantitation of TIMP1, MMP1, and MMP9 released by HDFs by qRT-PCR. *n* = 3, *P < 0.05, ***P < 0.001, ****P < 0.0001. **k** Western blot analysis showing the change of COL3, TIMP1, MMP1 and MMP9 in HDFs. **l** Quantitative of COL3, TIMP1, MMP1, and MMP9 in HDFs by western blot. *n* = 3, *P < 0.05, **P < 0.01, ***P < 0.001. **m** Quantitation of IL-1β, IL-6, and TNF-α released by HDFs by qRT-PCR. *n* = 3, *P < 0.05, **P < 0.01, ***P < 0.001, ****P < 0.0001. **n** Quantitation of IL-1β, IL-6, and TNF-α released by HDFs was detected through ELISA. *n* = 3, **P < 0.01, ****P < 0.0001

The dermis, the second layer of the skin beneath the epidermis, is also affected by UVB. Fibroblasts are one of the important cells in the dermis with functions such as secretion of collagen and MMPs that affect the ECM and ultimately the appearance of the skin such as wrinkles.^30^ To study the effects of UVB on the dermis, we used HDFs, which produce multiple ECM proteins that are essential for skin structure and function. There was no significant difference in PKH26-labeled EV that were up-taken by HDFs whether in the control group or under UVB irradiation (Fig. 3a and Supplementary Fig. 2d). We found that UVB irradiation caused similar changes in HDFs as in HaCaTs, such as increased ROS levels (Fig. 3b, c), senescence (Fig. 3d, e and Supplementary Fig. 2i, j), DNA damage (Fig. 3f, g), inflammation (Fig. 3m, n), migration (Supplementary Fig. 2e–h) and reduced proliferation (Fig. 3h), which were all alleviated by EV treatment.

Moreover, we investigated the impact of UVB and EV on collagen synthesis, which is a key process in maintaining skin elasticity and integrity. Matrix metalloproteinase (MMP) and the tissue inhibitor of metalloproteinase (TIMP) play crucial roles in preserving extracellular matrix (ECM) homeostasis that is directly related to wrinkles and sagging in skin appearance.^31^ We found that UVB irradiation decreased TIMP1 and COL3 levels while increasing MMP1 and MMP3 levels (Fig. 3i–l), indicating a disruption of collagen homeostasis and degradation of collagen fibers. In contrast, EV treatment restored the balance of TIMP1, MMP1, MMP3, and COL3 levels (Fig. 3i–l), suggesting a protective role of EV against UVB-induced collagen damage. These results indicate that UVB irradiation induces oxidative stress, DNA damage, senescence, inflammation, and impaired collagen synthesis in HDFs, the main features of photoaging in the dermis. Remarkably, treatment with EV demonstrates a capacity to mitigate these effects, thereby preserving the function and structure of the dermal layer.

Collectively, our in vitro study demonstrates that AMSC-EV and HUMSC-EV can protect both epidermal and dermal cells from UVB-induced photoaging by modulating various cellular processes.

### AMSC-EV and HUMSC-EV mitigated photoaging in a reconstructed full-thickness skin model

**Fig. 4:**
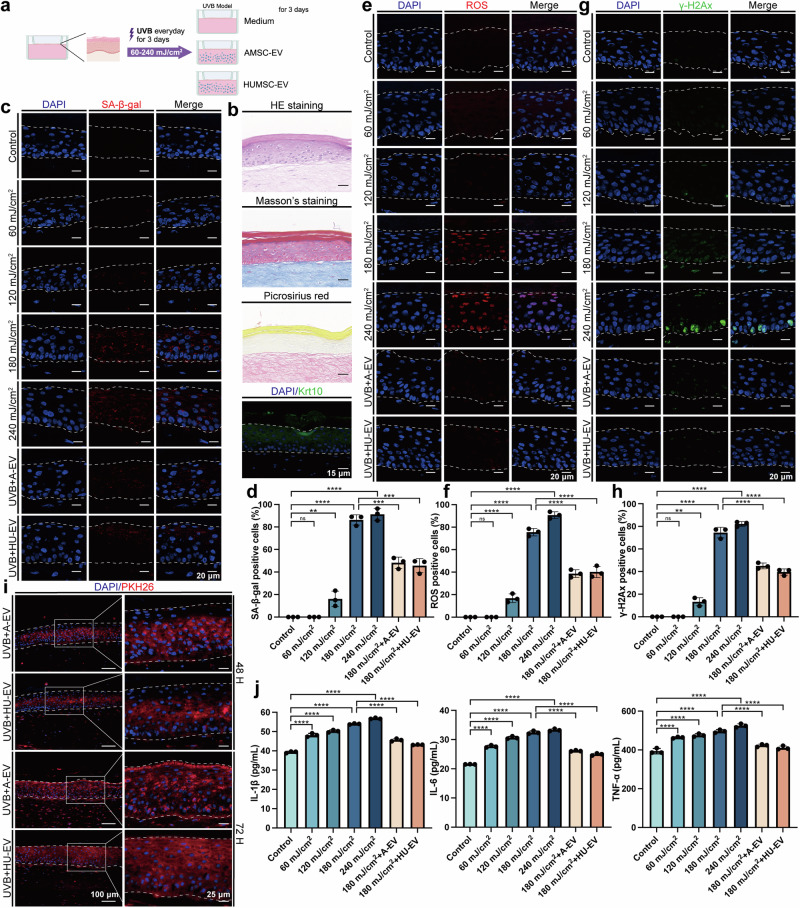
AMSC-EV and HUMSC-EV mitigated photoaging in constructed full-thickness skin model. **a** Schematic representation of establishment and treatment of T-Skin model (Created with BioRender.com). **b** Representative images of histological analysis and biomarker staining of the T-Skin model (scale bar, 15 μm). **c** Representative images of SA-β-gal staining (scale bar, 20 μm). **d** Quantitation of SA-β-gal positive cells. *n* = 3, **P < 0.01, ***P < 0.001, ****P < 0.0001. **e** Representative images of ROS staining (scale bar, 20 μm). **f** Quantitation of ROS-positive cells. *n* = 3, ****P < 0.0001. **g** Representative images of γ-H2Ax staining (scale bar, 20 μm). **h** Quantitation of γ-H2Ax positive cells. *n* = 3, **P < 0.01, ****P < 0.0001. **i** Representative images of MSC-EV uptake by T-Skin model after 48 and 72 h (left, scale bar, 150 μm; right, scale bar, 25 μm). **j** Quantitation of IL-1β, IL-6, and TNF-α released by T-Skin model was detected through ELISA. *n* = 3, ****P < 0.0001

The protective effects of AMSC-EV and HUMSC-EV in keratinocyte and fibroblast cell models prompted us to investigate further their role in an in vitro T-Skin photoaging model (Fig. 4a). T-Skin is a commercialized reconstructed full-thickness skin model, which shares some similarities with normal human skin in structure and biomarkers (Fig. 4b). Following various doses of UVB irradiation, we observed that 180mJ/cm^2^ every day for 3 days of irradiation induced a senescence phenotype, as evidenced by the increased presence of senescence (Fig. 4c, d), enhanced oxidative stress (Fig. 4e, f) and DNA damage (Fig. 4g, h). We added PKH26-stained EV and found that partial uptake occurred after 48 h, and EV uptake was completed in the whole model after 72 h (Fig. 4i). The addition of EV to the photoaging T-Skin model significantly alleviated SA-β-gal levels, ROS levels, and γ-H2Ax levels (Fig. 4c–h). Moreover, senescence markers P16 and LMNB1 were also rescued after EV stimulation (Supplementary Fig. 4a, b). UVB is a pivotal trigger of skin inflammation, which leads to the causation and exacerbation of numerous skin diseases.^32^ We then measured the expression levels of pro-inflammatory cytokines IL-1β, IL-6, and TNF-α using ELISA and found that UVB irradiation increased their secretion in the T-Skin model, and treatment with EV decreased their secretion, indicating an anti-inflammatory effect of AMSC-EV and HUMSC-EV (Fig. 4j).

Taken together, these results suggested that AMSC-EV and HUMSC-EV significantly ameliorated their photoaging oxidative stress and DNA damage levels while also alleviating their secretion of inflammatory factors in skin organoids.

### The therapeutic effect of AMSC-EV and HUMSC-EV in nude mice model of photoaging

**Fig. 5:**
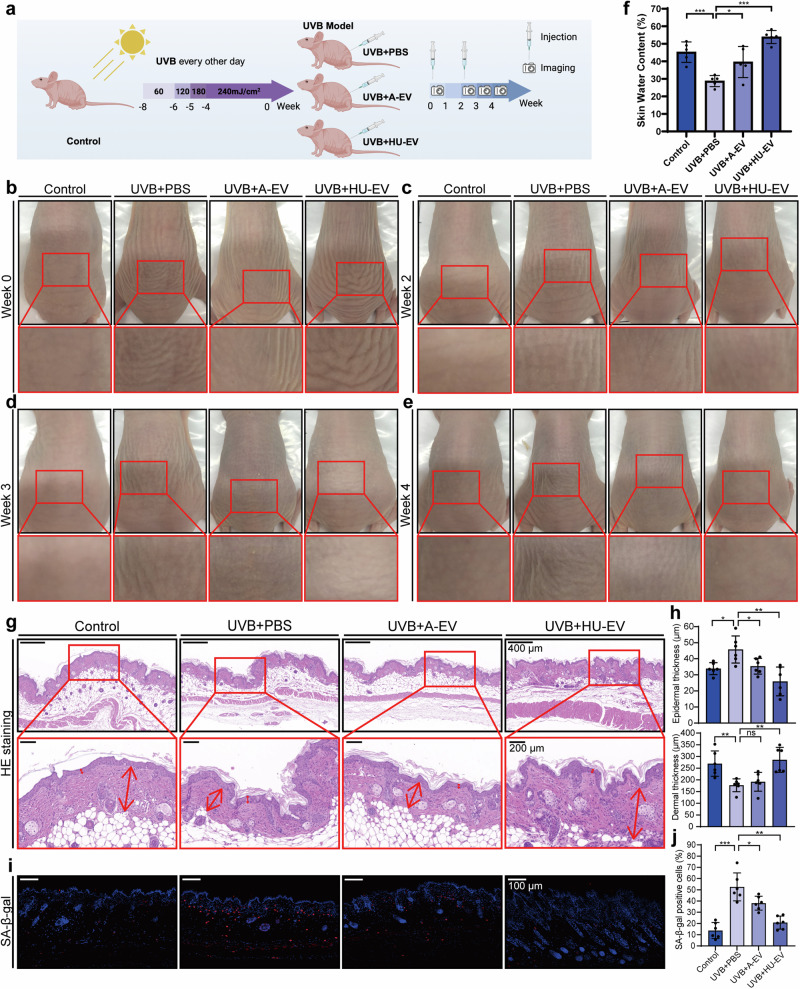
Histological analysis of the dorsal skin in nude mice after UVB irradiation and EV treatment. **a** Schematic description of the establishment and treatment of the photoaging model (Created with BioRender.com). **b**–**e** Representative images of mouse dorsal skin. From left to right: control group, UVB-exposed groups treated with PBS, AMSC-EV, and HUMSC-EV. **f** Transepidermal water loss content. *n* = 5, *P < 0.05, ***P < 0.001. **g** Representative images of HE staining (top, scale bar, 400 μm; bottom, scale bar, 200 μm). **h** Epidermal and dermal thickness analysis. *n* = 6, *P < 0.05, **P < 0.01. **i** Representative images of SA-β-gal staining. **j** Quantitation of SA-β-gal positive cells. *n* = 6, **P < 0.01, ***P < 0.001

The promising outcomes of AMSC-EV and HUMSC-EV in the in vitro cellular and T-Skin model motivated us to conduct additional investigation into their effect on skin photoaging in vivo. We established a photoaging model as previously reported^30^ in which UVB was administered every other day for 8 weeks in nude mice. Mice were randomly divided into four groups: control group and UVB exposure groups, which were separately treated with PBS, AMSC-EV, or HUMSC-EV (Fig. 5a). After establishing the UVB irradiation model, we captured images and investigated the wrinkle formation of the back skin of mice at week 0, 2, 3, and 4. At week 0, there were almost no wrinkles in the control group. In contrast, the UVB-treated groups exhibited deep and wide wrinkles, indicating the successful establishment of the photoaging model (Fig. 5b). Remarkably, from the second week, there were significantly fewer and thinner wrinkles in the EV-treated group (Fig. 5c–e). We investigated transepidermal water loss (TEWL) to characterize skin barrier function. Excess moisture content evaporated from the skin after UVB irradiation, and moisture loss was significantly reduced in EV-treated groups (Fig. 5f).

Further, histological analysis was performed to evaluate the skin condition and treatment effect. Hematoxylin and eosin (HE) staining was utilized to show the alterations in skin structure. Compared to the control group, UVB irradiation caused thicker epidermis and thinner dermis, and EV treatment significantly recovered thickness changes (Fig. 5g, h). Additionally, the number of SA-β-gal positive cells significantly increased after UVB irradiation and decreased significantly after EV-treated (Fig. 5i, j). Senescence markers P16 and LMNB1 were also restored after EV stimulation (Supplementary Fig. 5a, b). The above results indicate that EV can effectively mitigate the detrimental effects of UVB radiation on skin structure and aging.

**Fig. 6:**
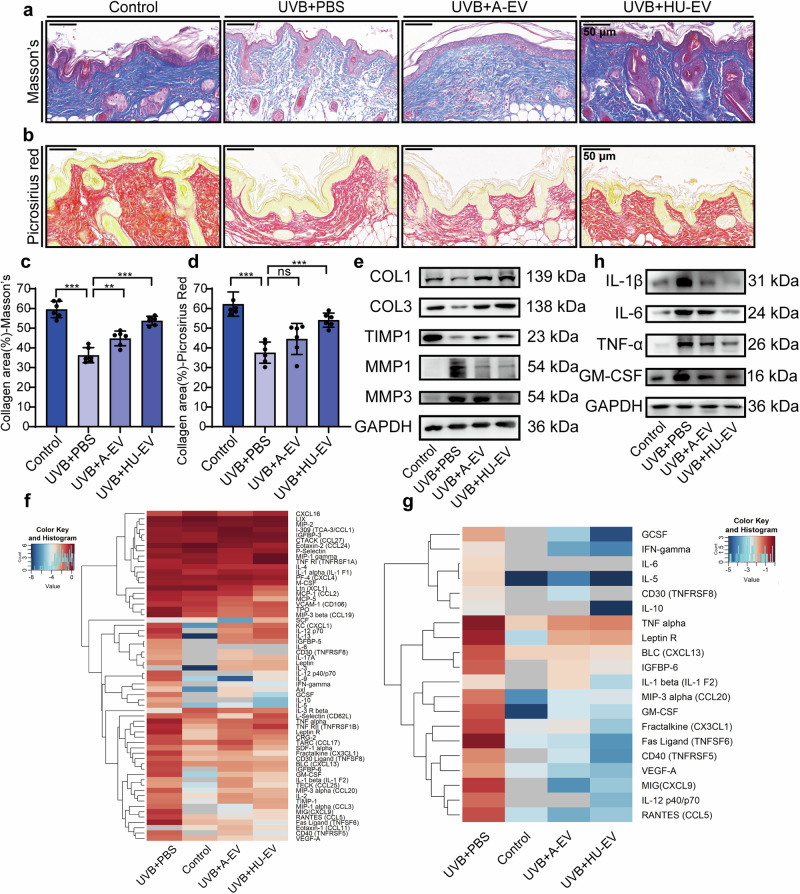
Histological analysis and inflammation antibody array of the dorsal skin in nude mice after UVB irradiation and EV treatment. **a** Representative images of Masson’s staining (scale bar, 50 μm). **b** Representative images of picrosirius red staining (scale bar, 50 μm). **c** Quantitation of collage area from Masson’s staining statistics. *n* = 6, **P < 0.01, ***P < 0.001. **d** Quantitation of collage area from picrosirius red staining statistics. *n* = 6, ***P < 0.001. **e** Western blot analysis for COL1, COL3, TIMP1, MMP1 and MMP3. **f** The top inflammatory factors examined by Inflammation Antibody Array. **g** Heatmap highlighting the representative inflammatory factors examined by the Inflammation Antibody Array. **h** Western blot analysis for inflammatory factors IL-1β, IL-6, TNF-α, and GM-CSF

Skin histology analysis could elucidate the amount of collagen deposition. Both Masson’s staining and picrosirius red staining were carried out to visualize alterations in dermal collagen levels. UVB exposure led to a reduction in the total amount of collagen. Compared with the control group, there were abundant and dense collagen fibers in the AMSC-EV and HUMSC-EV groups (Fig. 6a, c). Similarly, picrosirius red staining confirmed these findings (Fig. 6b, d), showing enhanced collagen restoration following EV treatment. Additionally, we performed Verhoeff’s Van Gieson staining to evaluate changes in elastin and found EV treatment restored elastin levels (Supplementary Fig. 5c, d).

We then analyzed collagen proteins and factors associated with dermal matrix remodeling. We found a significant decrease in the protein expression levels of COL1 and COL3, a significant increase in the levels of MMP1 and MMP3, and a significant reduction in the level of TIMP1 after UVB irradiation. These changes were restored after the addition of EV. (Fig. 6e). Additionally, we evaluated the expression of inflammatory factors by Inflammation Antibody Array (Fig. 6f). We show the results of representative inflammatory factor changes, including GCSF, IFN-γ, IL-6, and others (Fig. 6g). UVB exposure increased the expression of inflammatory factor, while treatment effectively restores these factor levels. After treatment, representative Western blot results confirmed the reduction trend in inflammatory factors, including IL-1β, IL-6, TNF-α, and GM-CSF (Fig. 6h).

Collectively, these findings demonstrate that UVB exposure induces skin changes in appearance, function, and histology, accompanied by inflammatory responses in vivo, and MSC-EV reversed these changes in a positive direction.

In addition, the observed therapeutic effect of HUMSC-EV seems better than that of AMSC-EV, which warrants further investigation.

### MSC-EV rescues HDFs and HaCaTs photoaging by transferring TIMP1

**Fig. 7:**
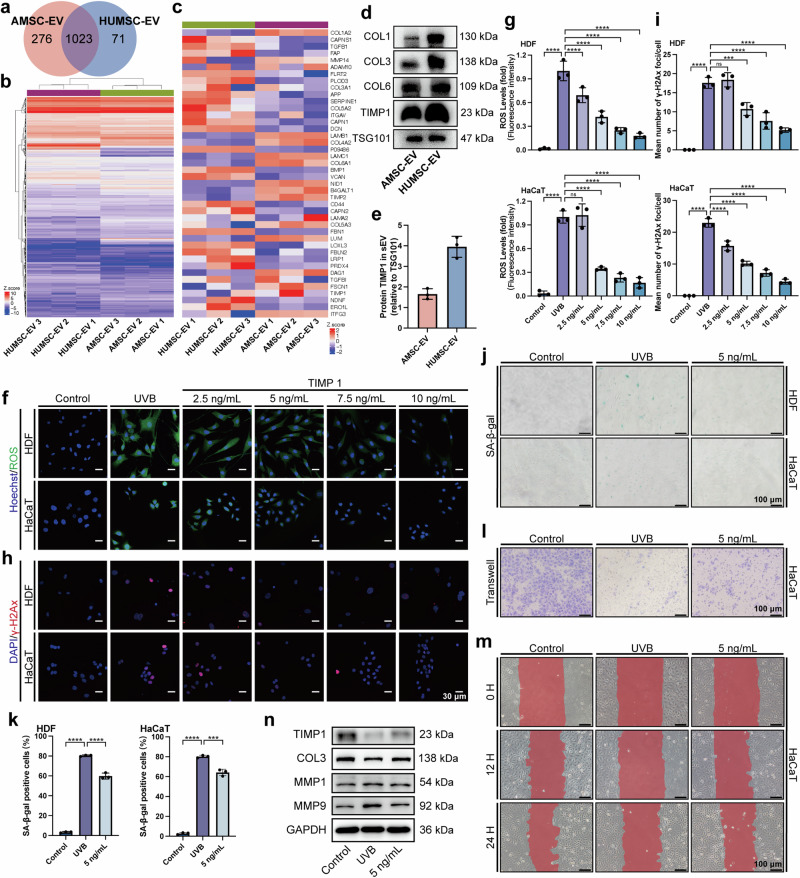
MSC-EV rescues HDFs and HaCaTs photoaging by upregulating TIMP1. **a** Venn diagram of the protein contents of AMSC-EV and HUMSC-EV. **b** Heatmap of the protein contents of AMSC-EV and HUMSC-EV. **c** Heatmap of extracellular matrix organization. **d** Western blot analysis showing COL1, COL3, COL6, and TIMP1 expression in AMSC-EV and HUMSC-EV. **e** Quantitation of TIMP1 in AMSC-EV and HUMSC-EV was detected by Western blot. *n* = 3. **f** Representative immunofluorescence staining images of positive cells of ROS (green) and DAPI (scale bar, 35 μm). **g** Quantitation of ROS-positive cells. *n* = 3, ****P < 0.0001. **h** Representative immunofluorescence staining images of positive cells of γ-H2Ax (red) and DAPI (scale bar, 20 μm). **i** Quantitation of a mean number of γ-H2Ax foci/cell. *n* = 3, ***P < 0.001, ****P < 0.0001. **j** Representative images of SA-β-gal staining in HDFs and HaCaTs (scale bar, 100 μm). **k** Quantitation of SA-β-gal positive cells in HDFs and HaCaTs. *n* = 3, ***P < 0.001, ****P < 0.0001. **l** Representative images of transwell assays of HaCaTs (scale bar, 100 μm). **m** epresentative images of migration assay of HaCaTs (scale bar, 100 μm). **n** Western blot analysis showing the change of COL3, TIMP1, MMP1 and MMP9 in HDFs

Since both AMSC-EV and HUMSC-EV exhibited protective effects against photoaging, we speculate that there should be shared components within their cargo responsible for this outcome. As a crucial component of EV cargo, proteins are known to exert significant influence on EV functions. We first performed a protein analysis of MSC-EV. Overall, we detected 1,370 proteins, of which 1023 proteins were common to AMSC-EV and HUMSC-EV (Fig. 7a).

Gene ontology (GO) analysis showed that proteins that are highly expressed in both AMSC-EV and HUMSC-EV are mainly enriched in extracellular matrix organization (Fig. 7b, c), suggesting their potential impact on extracellular matrix remodeling mediated by the shared highly expressed proteins. We performed Western blot for the highly expressed TOP10 proteins (COL1A2, DCN, COL3A1, COL6A1, TIMP1, FSTL1, LUM, NID1, PTX3, and ENO1). The levels of TIMP1, COL1A2, COL3A1, and COL6A1, closely associated with extracellular matrix homeostasis, are all high in AMSC-EV and HUMSC-EV (Fig. 7d, e and Supplementary Fig. 6a). NID1 and FSTL1, which may be related to extracellular matrix homeostasis, are also highly expressed (Supplementary Fig. 6b, c).

We further analyzed TIMP1 due to its well-established role in ECM remodeling^27,33^ and potential involvement in the photoaging process. In aged mice skin, we observed a decrease in TIMP1 protein expression compared to young mice (Supplementary Fig. 6d, e). Silence of TIMP1 by siRNAs in HaCaTs and HDFs resulted in a significant increase in SA-β-gal positive cells (Supplementary Fig. 6f, g) and expression of senescence-associated markers (Supplementary Fig. 6h–k), indicating a crucial role of TIMP1 in the aging process. To investigate whether EV exert protective effects against photoaging by transferring TIMP1, we first added TIMP1 into the cell culture medium according to the concentration gradient. We found that 5 ng/mL of TIMP1 was able to reduce UVB-induced oxidative stress (Fig. 7f, g) and DNA damage (Fig. 7h, i). Assays for ROS and γ-H2Ax were also performed on days 1, 2, and 3 after the addition of 5 ng/mL of TIMP1, and remarkable reductions were observed on day 3 (Supplementary Fig. 7a–d). Other hallmarks of photoaging, including elevated SA-β-gal positive cells in the HDFs and HaCaTs (Fig. 7j, k), reduction of HaCaTs migration ability (Fig. 7l, m and Supplementary Fig. 7e, f), as well as extracellular matrix degradation (Fig. 7n and Supplementary Fig. 7g), were all inhibited after TIMP1 treatment. In addition, a significant decrease in the levels of MMP1, MMP3, and MMP9 after TIMP1 treatment was observed (Supplementary Fig. 8a–f).

Taken together, we demonstrated that TIMP1 is a pivotal protein in EV and plays a crucial role in preventing photoaging, particularly in oxidative stress, DNA damage, cell migration, and extracellular matrix remodeling.

### The therapeutic effect of MSC-EV is mediated through the downregulation of Notch signaling pathway

**Fig. 8:**
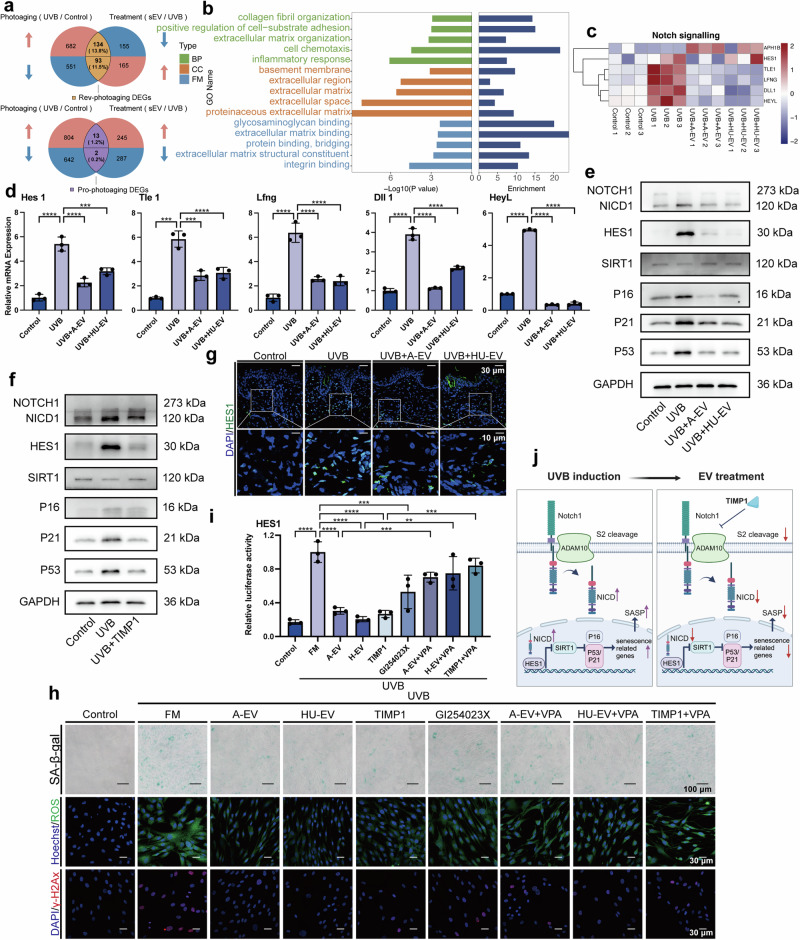
MSC-EV rescues HDFs photoaging by downregulating NOTCH signaling pathway. **a** Venn diagram showing the overlapped genes between photoaging (UVB/Control) and Treatment (EV/UVB) based on data from RNA-seq. Rev-photoaging-DEGs were defined as subsets of overlapped DEGs that were changed in the opposite direction in photoaging and treatment. **b** Representative GO terms of Rev-photoaging DEGs for Biological Process (green), Cellular Component (orange), and Molecular Function (blue) were shown. **c** Heatmaps showing the expression profile of genes in response to UVB and EV treatment in Notch signaling. **d** Quantitation of different mRNA levels of Hes1, Tle1, Lfng, Dll1, and HeyL. **e** Western blot analysis showing expression of NOTCH1, NICD1, HES1, SIRT1, P16, P21, P53 and GAPDH after EV treatment. **f** Western blot analysis showing expression of NOTCH1, NICD1, HES1, SIRT1, P16, P21, P53 and GAPDH after TIMP1 treatment. **g** Representative immunofluorescence staining images of positive cells of HES1 in nude mice dorsal skin injected PBS, AMSC-EV, or HUMSC-EV (top, scale bar, 30 μm; bottom, scale bar, 10 μm). **h** Representative immunofluorescence staining images of SA-β-gal (scale bar, 100 μm), ROS (green), and γ-H2Ax (red) (scale bar, 30 μm). **i** Luciferase-reporter assays of HES1 transcriptional activity in HDFs after UVB, FM (culture medium), EV, TIMP1, GI254023X, and VPA treatment. *n* = 3, **P < 0.01, ***P < 0.001, ****P < 0.0001. **j** Schematic of the role of EV in mediating UVB-induced photoaging of Notch signaling and cellular senescence. (Created with BioRender.com)

To investigate the impact of MSC-EV on signaling pathways, we performed RNA sequencing (RNA-seq) analysis on HDFs control, UVB exposure, and treatment with AMSC-EV and HUMSC-EV groups, and analyzed different expression genes (DEGs) overlapped in both AMSC-EV and HUMSC-EV treatment groups. DEGs up-regulated by UVB but repressed by EV and DEGs downregulated by UVB but restored by EV were identified, resulting in a set of Rev-photoaging DEGs, while genes that changed in the same direction were categorized as Pro-photoaging DEGs (Fig. 8a). We observed a significantly higher count of Rev-photoaging DEGs compared to Pro-photoaging DEGs. GO analysis indicated that the Rev-photoaging DEGs were enriched in ECM degradation and immune responses (Fig. 8b). Further investigation into altered signaling pathways unveiled that EV downregulated the UVB-induced genes in Notch signaling pathway (Fig. 8c). Subsequent qPCR analysis confirmed the changes in related molecules Hes1, Tle1, Lfng, Dll1 and HeyL (Fig. 8d).

The Notch signaling pathway is an important signaling pathway vital to cell fate, proliferation, and tissue homeostasis. The NOTCH receptor is first cleaved in the Golgi and translocated to the cell membrane. The Notch receptor is activated by binding to ligands presented by neighboring cells, such as Delta-like 1 (DLL1), which promotes a conformational change that exposes Notch to cleavage by A disintegrin and metalloproteinases (ADAMs) at the S2 site. The remainder of the NOTCH receptor is further cleaved to form the Notch intracellular domain (NICD) by γ-secretase at the cell membrane. NICD can translocate to the nucleus, and transcriptional activation of Notch target genes is initiated, such as those of the Hes and Hey family.^34^ Transducin-like Enhancer of Split 1 (TLE1) is a multitasked transcriptional corepressor that acts through the Notch signaling pathway.^35^ Lunatic Fringe (Lfng), the Notch modulator gene, is mainly involved in intercellular coupling and delays intercellular Notch signaling transmission.^36^ Hes1, a crucial downstream molecule of the Notch signaling pathway, prompted us to explore its role in EV-mediated alleviation of photoaging. Literature reports suggest that p16, p21, and p53 are downstream molecules of HES1, which further influence the release of SASP.^37,38^ In UVB-induced HDFs, western blot analysis demonstrated that changes in Notch1 and its downstream genes HES1, SIRT1, P16, P21, and P53 were all recovered by AMSC-EV and HUMSC-EV (Fig. 8e and Supplementary Fig. 9a). Additionally, we found that TIMP1 exerted a similar role in mitigating the activation of the NOTCH signaling pathway (Fig. 8f and Supplementary Fig. 9b). Furthermore, immunofluorescence staining showed that AMSC-EV and HUMSC-EV exhibited much less HES1 in the photoaged skin in nude mice (Fig. 8g and Supplementary Fig. 9c).

To determine whether EV and TIMP1 modulate downstream pathways by inhibiting Notch signaling, we introduced Valproic Acid Sodium (VPA), a Notch pathway activator. Following VPA addition in HDFs, we observed a reduction in the efficacy of EV and TIMP1 in mitigating senescence, clearing ROS, and repairing DNA damage (Fig. 8h and Supplementary Fig. 9d–f). ADAM10, an essential substrate for Notch activation, is a proposed target of TIMP1. We then employed GI254023X, an ADAM10 inhibitor, and found that it replicated the photoaging alleviation effects observed with TIMP1 (Fig. 8h and Supplementary Fig. 9d–f). Similar findings were also observed in the T-skin model. As demonstrated in Supplementary Fig. 10a–d, the addition of Notch activator VPA decreased the efficacy of EV and TIMP1, while ADAM10 inhibitor GI254023X partially recapitulated the effects of EV and TIMP1. Additionally, it inhibited the UVB-induced upregulation of HES1 transcriptional activity and protein expression (Fig. 8i and Supplementary Fig. 10e, f).

In summary, these results indicate that EV and its content TIMP1 may exert their effects by inhibiting the Notch signaling pathway through TIMP1-mediated suppression of ADAM10 (Fig. 8j).

## Discussion

UV, a potent human carcinogen, induces skin cancer in the absence of additional initiators or promoters.^39^ Excessive exposure to UV rays, mainly to UVB, triggers erythema, an acute skin inflammatory reaction associated with redness, itching, and pain. It also leads to DNA damage, gene mutations, immunosuppression, oxidative stress, and inflammation, leading to skin diseases and even skin cancer.^40,41^ Mitigating UVB-induced photoaging can help alleviate associated oxidative stress and inflammatory responses, preventing skin diseases. With the increasing demand for cosmetic enhancement, substantial efforts have been made to rescue skin photoaging. In recent years, EV have received considerable attention due to their high therapeutic potential in tissue repair. EV derived from three-dimensional spheroids HDFs have been reported to significantly reduce MMP levels and enhance collagen expression in HDFs.^30^ mRNA-encapsulating EV delivered intradermally, as reported by Yi You et al., have been reported to be used in improving photoaged skin wrinkles, showing favorable therapeutic effects.^11^ In this study, we focused on MSC-EV, which have been extensively studied in the realm of conditions such as skin wound healing. However, their protective properties against photoaging remain a relatively less explored area. Our work presents a comprehensive demonstration of the therapeutic potential of MSC-EV, substantiated by rigorous data across cellular, tissue-like, and in-vivo murine models, marking a significant advancement in the field. Moreover, in addition to investigating the therapeutic potential, we explored the preventive effects of MSC-EV. As demonstrated in Supplementary Fig. 11, pre-treatment with MSC-EV prior to UV exposure significantly reduced ROS and γ-H2Ax levels in HaCaTs and HDFs. Further studies are needed to explore MSC-EV as a preventive strategy against diseases such as photoaging or skin cancer resulting from UVB exposure. We showed that both AMSC-EV and HUMSC-EV can ameliorate photoaging, and in the nude mice model, the observed therapeutic effect of HUMSC-EV seems better than that of AMSC-EV (Fig. 5). As a therapeutic agent, AMSC-EV and HUMSC-EV have their own advantages and disadvantages in terms of their tissue origin. AMSC-EV can be processed from adipose tissue, yet the extraction procedures are inherently invasive, posing a challenge. Nonetheless, patients have the potential to utilize their own adipose tissue. Conversely, HUMSC-EV are derived from umbilical cord tissue, which is a relatively easily accessible source. However, in the absence of stored umbilical cord-derived MSCs from the patients themselves, alternative donor sources become the only viable option. In addition, MSCs from different tissues have different angiogenic, inflammatory, and matrix-remodeling potential properties.^42^ For example, both BM-MSC and UCT-MSC could promote lung recovery in experimental bronchopulmonary dysplasia, but UC-MSC suppresses lung macrophage infiltration and promotes epithelial cell healing to a greater degree.^43^ Therefore, the subtle variations in therapeutic effects observed between AMSC-EV and HUMSC-EV in our study might be contributed to the distinct biological characteristics of MSC, as EV partially recapitulate the properties of their parental cells. Further investigation is warranted to elucidate the variances among MSC-EV originating from distinct sources.

MSC-EV exerts their effects by transferring cargo components into target cells. In our study, we found both AMSC-EV and HUMSC-EV exhibited protective effects against photoaging, prompting us to investigate the shared proteins as potential underlying mechanisms. We identified TIMP1 as a key player, a natural inhibitor of MMPs that is involved in the tissue remodeling of the ECM. The balance between MMP and TIMP1 activities is critical in normal and pathological events such as wound healing, tissue remodeling, and angiogenesis.^44^ We found that AMSC-EV and HUMSC-EV are enriched with TIMP1, which can mimic the effects of EV in mitigating ROS, reducing DNA damage, and decreasing ECM degradation. The role of TIMP1 in ECM remodeling is well known, but here, we highlight its significant role in aging. We observed that TIMP1 levels decreased in aging mice dorsal skin. Knockdown of TIMP1 resulted in cellular senescence in the epidermal keratinocytes and dermal fibroblasts. Moreover, we demonstrated that TIMP1 could inhibit ADAM10, a key substrate for Notch activation, and then downregulate the Notch signaling pathway. The Notch signaling pathway in the skin plays a critical role in regulating cell proliferation and differentiation, thereby influencing epidermal homeostasis.^25^ Anna Mandinova et al. reported that the Notch signaling pathway was a protective anti-apoptotic mechanism in response to UVB in keratinocytes.^45^ Lanying Liu et al. found that the inhibition of Notch2 reduced UVB-induced damage in retinal pigment epithelial cells.^46^ However, it remains elusive how Notch signaling changes during skin photoaging, and what impact EV treatment has on it. To the best of our knowledge, our research is the first to show that AMSC-EV and HUMSC-EV rescued skin photoaging by downregulating UVB-induced Notch elevation. Our study provides new insights into the role of TIMP1 in aging and highlights the potential anti-photoaging effects of AMSC-EV and HUMSC-EV. However, we could not exclude the involvement of other proteins. Additionally, miRNA is another choice, which is of particular interest for understanding the molecular mechanisms of EV’s mode of action. We also performed miRNA sequencing of AMSC-EV and HUMSC-EV, identifying 399 shared miRNAs. The top 20 expressed miRNAs are shown in Supplementary Fig. 12, and some of these miRNAs have been reported to have anti-aging functions. For example, miR-99a-5p from macrophage-derived EV could regulate follicular activation and improve ovarian function in old mice by modulating the local environment,^47^ while miR-199a-3p mimic significantly suppressed increased endothelial senescence under diabetic conditions.^48^ Further investigation is needed to validate the effects of these miRNAs and other proteins. In the future, we plan to optimize MSC-EV to achieve elevated expression levels of TIMP1 or other useful molecules, which will be combined with innovative technologies such as microneedle or needle-free injection systems to enhance its anti-photoaging effect.

MSC-EV possess multiple capabilities that contribute to their therapeutic potential, including anti-inflammation, immunomodulation, and the promotion of angiogenesis. In the context of photoaging, our study unveils an additional facet of MSC-EV, showcasing their robust ECM remodeling capacity. The ECM of the skin plays a pivotal role in maintaining its integrity, serving as the foundational structure for skin support and resilience and constantly remodels to affect tissue homeostasis. Amanpreet Kaur et al. reported that the aged ECM promotes melanoma cell metastasis and inhibits T cell migration.^49^ Ishier Raote et al. found that regulating ECM components may have therapeutic effects in wound healing and fibrosis processes.^50^ We showed that AMSC-EV and HUMSC-EV significantly increased dermal collagen and elastin levels in photoaged skin. Here is an interesting phenomenon. UVB rays are known to primarily impact the epidermis due to their shallow penetration depth. However, we observed significant changes in the dermis after UVB radiation when we injected MSC-EV subcutaneously but found improvement in the epidermis. Therefore, we speculate that the intricate crosstalk between the epidermis and dermis might explain the effects of UVB and MSC-EV. For example, UVB radiation induces an inflammatory response in the epidermis, leading to the production of pro-inflammatory cytokines and chemokines such as IL-6, IFN-γ and IL-1β.^51,52^ These inflammatory mediators can penetrate into the dermis, activating immune cells and fibroblasts and contributing to dermal changes. EV-mediated delivery of Gstm2 mRNA has been reported to promote dermal fibroblasts to regulate skin epidermal cell function by paracrine secretion of NACA.^53^

Additionally, our study observed that MSC-EV and TIMP1 reduced ROS levels and DNA damage, which presents an intriguing question regarding the underlying mechanisms responsible for this reduction. One plausible explanation could be that MSC-EV and TIMP1 may modulate signaling cascades that regulate ROS production and DNA damage response pathways. For example, UVB irradiation induces mitochondrial DNA (mtDNA) loss, thereby triggering mitochondrial dysfunction, generating ROS, causing DNA damage, and ultimately contributing to photoaging.^54^ Studies by Li et al. have shown that EV derived from neural stem cells can improve mitochondrial function and restore the normal distribution of mitochondrial biogenic factors such as PGC1α, NRF1, and COXIV.^55^ Additionally, Xiao et al. reported that reducing TIMP1 levels increases ROS levels.^56^ Based on these findings, we hypothesize that UVB-induced mtDNA loss may initiate mitochondrial dysfunction, ROS generation, DNA damage, and photoaging. MSC-EV and TIMP1 may potentially enhance mitochondrial function, mitigate ROS production, and reduce DNA damage. Further investigations are warranted to elucidate the precise mechanisms underlying these observations.

**Fig. 9:**
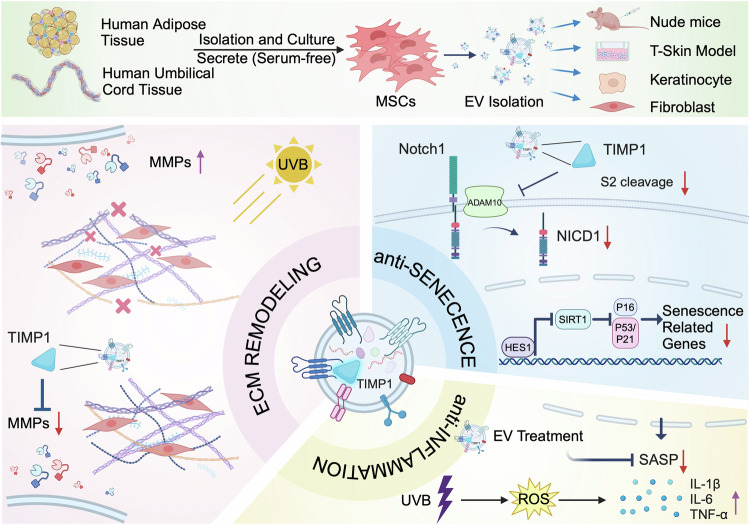
Schematic illustrations of AMSC-EV and HUMSC-EV repairments on photoaging in vitro and in vivo via regulation of the TIMP1/Notch pathway (Created with BioRender.com). AMSC-EV and HUMSC-EV improve photoaging in UVB-irradiated nude mice, HaCaTs, HKCs, HDFs, and T-Skin models through anti-inflammatory effects, ECM remodeling, and anti-senescence properties. Mechanistically, proteomic analysis revealed that TIMP1 is highly expressed in both AMSC-EV and HUMSC-EV and exerts similar effects to MSC-EV. TIMP1 inhibits MMPs, thereby altering extracellular matrix remodeling. Additionally, TIMP1 downregulates the activated Notch pathway and inhibits its downstream targets Hes1, P16, P21, and P53

In summary, our findings highlight the therapeutic effect of MSC-EV at cellular, tissue-like, and in-vivo murine models through anti-inflammatory effects, ECM remodeling, and anti-senescence properties (Fig. 9). Mechanistically, proteomic analysis revealed that TIMP1 is highly expressed in both AMSC-EV and HUMSC-EV and exerts similar effects to MSC-EV. TIMP1 plays crucial roles in mediating the therapeutic effects of MSC-EV, with TIMP1 contributing to the alleviation of cellular senescence, DNA damage, oxidative stress, and ECM hydrolysis, and subsequently downregulating Notch expression.

## Materials and methods

### Cell culture

Human adipose tissues and umbilical cords were obtained according to procedures approved by the Ethics Committee at Chinese Academy of Medical Sciences and Peking Union Medical College. MSCs were isolated from human adipose tissues and umbilical cords of healthy volunteers and culture-expanded as previously reported.^21^

HDF cells were purchased from ATCC and maintained in Fibroblast Medium (ScienCell, 2301). HaCaTs were obtained from the Cell Resource Center, Peking Union Medical College (which is the headquarter of National Science & Technology Infrastructure—National BioMedical Cell-Line Resource, NSTI-BMCR) and maintained in minimum essential medium with 10% fetal bovine serum, 100 U/mL penicillin and 100 μg/mL streptomycin. Cells were maintained in a 37 °C humidified incubator with 5% CO_2_ and passaged with trypsin/EDTA.

Human primary keratinocytes (HKCs) were purchased from iCell Bioscience Inc. according to the manufacturer, HKCs were isolated from foreskins. They were cultured in iCell Primary Keratinocyte Media (iCell Bioscience, PriMed-iCELL-010) with Primary Keratinocyte Culture Supplement. Cells were maintained in a 37 °C humidified incubator with 5% CO_2_ and passaged with trypsin/EDTA.

MSCs of passage 5, HDFs of passage 3 or 4, and HKCs of passage 2 or 3 were used in our experiments.

In all, 10 μM ADAM10 inhibitor GI254023X (HY-19956, MedChemExpress) were used in different experiments. After UVB, HDFs were incubated with ADAM10 inhibitor GI254023X (HY-19956, MedChemExpress) for 2 h, and then cells were incubated with fibroblast medium for an additional 24 h. After UVB, 2 mM Notch activator Valproic acid (Sodium Valproate) sodium (HY-10585A, MedChemExpress) were used with MSC-EV or TIMP1 for 24 h.

### Isolation of EV

We isolated MSC-EV following the MIEV2018 guidelines. After the AMSCs and HUMSCs reached 80–90% confluence, they were maintained in a serum-free medium for 48 h. The supernatants were collected and centrifuged at 800 × *g* for 5 min, followed by 3000 × *g* for 10 min to remove cells and debris. The supernatant was then filtered through a filter with 0.22 µm pore size fast flow polyethersulfone (PES) Express PLUS membrane (Millipore) to remove cellular debris and large vesicles and then concentrated with a 100 kDa MWCO cut-off membrane (Millipore). Following the employment of a MWCO cut-off membrane, the supernatant volume was decreased to under 5 mL from an initial volume of around 250 mL. Subsequently, the supernatant underwent ultracentrifugation at 110,000 × *g* for 70 min at 4 °C using a Beckman Coulter centrifuge. The resulting pellets were resuspended with PBS and ultra-centrifuged with 100 Ti Rotor for 70 min at 110,000 × *g* at 4 °C (Beckman Coulter).

### Transmission electron microscopy

The morphology of EV was visualized by TEM analysis. On a formvar/carbon-coated grid, 20 μL of freshly isolated EV were fixed in 1% glutaraldehyde in PBS (pH 7.4). Then negatively stained with 3% aqueous phosphotungstic acid for 5 min at room temperature in the dark. After the grid was dried for 10 min, EV were examined using TEM (Tecnai G2 Spirit TEM, Zeiss, Oberkochen, Germany) at 120 kV.

### Nanoparticle tracking analysis

According to the instructions, the size of AMSC-EV and HUMSC-EV was evaluated with Zeta View (Particle Metrix, Meerbusch, Germany). The samples were diluted to an appropriate multiple with 1 mL PBS and then were injected with a sterile syringe.

### ExoView analysis

Collected EV samples were analyzed using the ExoView platform (NanoView Biosciences, Boston, MA, USA) following the manufacturer’s procedure. Briefly, samples were dropped onto the chip and incubated to capture EV. Each chip is pre-coated with anti-C63, anti-CD81, anti-CD9, and MIgG-negative control antibodies. Chips were imaged with an ExoView R100 scanner after incubation with these antibodies. Data were analyzed using NanoViewer software.

### Mouse photoaging model and treatments

We purchased BALB/c-nu mice from the Laboratory Animal Center of Peking Union Medical School (Beijing, China). Animal use and experimental procedures were approved by the Animal Care and Use Committee of the Chinese Academy of Medical Sciences (ACUC-A02-2022-010). The nude mouse was irradiated with UVB (SIGMA High-tech, Shanghai, China) every other day for 8 weeks on dorsal skin. The minimal erythemal dose (MED) was 60 mJ/cm^2^. During the first 2 weeks, the irradiation intensity was set at 1 MED and was elevated to 2 MED (120 mJ/cm^2^) in the third week, to 3 MED (180 mJ/cm^2^) in the fourth week, and to 4 MED (240 mJ/cm^2^) during the fifth to eighth weeks. The total irradiated UVB volume was approximately 80 MED. Nude mice were randomly divided into 4 groups: (a) no UVB exposure (control); (b) UVB irradiation alone (UVB + PBS); (c) UVB irradiation with AMSC-EV (UVB + A-EV) and (d) UVB irradiation with HUMSC-EV (UVB + HU-EV). AMSC-EV or HUMSC-EV were subcutaneously injected into 10 different sites evenly on the whole dorsal skin using a BD insulin syringe (Ultra-Fine). The EV dose used was 200 μg per mouse. After a 4-week treatment, mice were sacrificed, and skin samples were harvested and fixed in 10% paraformaldehyde (PFA). Then, HE staining, Masson’s staining, and picrosirius red staining were carried out.

### Induction of cell photoaging

HaCaTs, HDFs, and HKCs were washed with PBS before exposure to UVB at the dose of 60 mJ/cm^2^/day for 3 days. Cells were then incubated with serum-free medium with or without EV at 200 ng/μL for another 24 h.

The T-Skin model was treated with UV every day for 3 days at a dose of 180 mJ/cm²/day and then medium with or without EV were applied for an additional 72 h.

### In vivo imaging

Isolated EV were labeled with PKH26 for in vivo Imaging. Briefly, PKH26 dye and EV diluted in diluent C were incubated together for 10 min, and staining was stopped with BSA. Unbound dye was removed by ultracentrifugation. PKH26-labeled EV were resuspended in PBS. In total, 100 μL PKH26-labeled EV (200 μg) were injected subcutaneously into ten different sites on the dorsal skin of nude mice using a BD insulin syringe (Ultra-Fine), and the fluorescence at 1 h and 24 h was acquired using the IVIS Spectrum In Vivo Imaging System (Caliper Life Sciences, Hopkinton, MA, USA). After treating mice with PKH26-labeled EV for 24, 48, and 72 h, the mice were sacrificed, and skin samples were harvested and fixed in 10% paraformaldehyde (PFA). The nuclei were then labeled with DAPI (Beyotime, Shanghai, China), and images were captured using an inverted microscope (Olympus, Tokyo, Japan).

### Quantitative real-time polymerase chain reaction(qRT-PCR)

TRIzol reagent (Invitrogen, Carlsbad, CA, USA) was used to extract the total RNA according to the manufacturer’s instructions. The concentration and purity of RNA were determined by optical density. M-MLV Reverse Transcriptase Kit (Takara, Tokyo, Japan) was used to synthesize cDNA. SYBR Green (YESEN, Shanghai, China) was used to perform Real-time PCR amplification, and relative mRNA expression was calculated by the 2^-ΔΔCt^ method and normalized to GAPDH expression.

### Western blotting

Proteins were extracted with radioimmunoprecipitation (RIPA) lysis buffer containing PMSF at a ratio of 100:1 between RIPA and PMSF. The concentration was measured using the BCA protein assay kit (Beyotime, Shanghai, China). GAPDH was used as an internal control. Then, it was detected with chemiluminescent detection (Tanon, Shanghai, China).

### Immunofluorescence staining

Cultured cells were fixed in ice-cold methanol at 4 °C for 10 min and rinsed the plate three times in phosphate-buffered saline (PBS) for more than 5 min each. Cells were permeabilized in 0.1% Triton X-100 at room temperature. The plate was rinsed with PBS three times, each more than 3 min. Non-specific binding was blocked by 1% bovine serum albumin (BSA) containing 0.5% Tween-20 in PBS for 60 min. Primary antibody incubations were performed overnight at 4 °C. The secondary antibody was incubated for 1 h at room temperature after rinse the plate with PBS for three times. The incubated cells were washed with PBS, and nuclei were labeled with DAPI (Beyotime, Shanghai, China). Co-staining of p16 and laminin B1 in T-Skin models or dorsal skin sections of nude mice was performed using the TSA Fluorescence Double Staining Kit (Abclone, Wuhan, China).

### Senescence-associated β-galactosidase staining

Cultured cells were washed in PBS, fixed in fixative solution at room temperature for 15 min, and rinsed on the plate three times. They were stained in freshly prepared β-Galactosidase Staining Solution (Beyotime, Shanghai, China) at 37 °C overnight. Images were taken with an inverted microscope (Olympus, Tokyo, Japan), and the percentages of positive regions were calculated and analyzed using ImageJ.

Paraffin-embedded tissue sections of T-Skin models or dorsal skin of nude mice injected with PBS (control) or MSC-EV were washed in PBS and fixed in fixative solution at room temperature for 15 min, followed by rinses. The sections were then permeabilized in 0.3% Triton X-100 at room temperature and rinsed three times with PBS, each rinse lasting more than 3 min. To block non-specific binding, the sections were incubated in 1% bovine serum albumin (BSA) containing 0.1% Triton X-100 in PBS for 60 min. Beta Galactosidase polyclonal antibody (Proteintech, Wuhan, China) incubations were performed overnight at 4 °C. After rinsing the sections with PBS three times, the secondary antibody was incubated for 1 h at room temperature. The nuclei were then labeled with DAPI (Beyotime, Shanghai, China), and images were captured using an inverted microscope (Olympus, Tokyo, Japan).

### Reactive oxygen species (ROS) detection

Intracellular ROS generation in HaCaTs and HDFs was measured by using the Reactive Oxygen Species Assay Kit (Beyotime, Shanghai, China). The cells were incubated with DCFH-DA for 30 min at 37 °C. Intracellular ROS generation in T-Skin models were measured by using Dihydroethidium (Beyotime, Shanghai, China). The models were incubated with Dihydroethidium for 30 min at 37 °C. These cells or models were washed with pre-warmed PBS, and nuclei were labeled with Hoechst.

### Transwell assay

We used 24-well transwell inserts with 8-μm pore size filters and 24-well culture plates (Corning, NY, USA). Cells were suspended in medium containing 3% FBS and plated 1 × 10^5^ cells per well in the upper chamber. 500 μL of medium containing 10% FBS was added in the lower chamber. After 12 h of incubation, cells on the upper surface of the filter membrane were removed with a cotton swab, and the migrated cells on the lower side of the filter membrane were stained with crystal violet.

### Migration assay

Cells were seeded in six-well plates and incubated at 37 °C to form a monolayer of cells. Cells were scraped with a p200 pipette tip and washed with PBS to remove floating cells. The HaCaTs were photographed after 0 h, 12 h, and 24 h. HDFs were photographed at 0 h, 6 h, and 12 h. The remaining width of the wound at the metering point was analyzed by ImageJ software. The migration rate was determined by the ratio of the closure area to the initial wound.

### Enzyme linked immunosorbent assay (ELISA)

HDFs, HaCaTs, or T-skin medium were collected and centrifuged at 3000 rpm for 20 min at 4 °C. The supernatant was collected, and IL-1β, IL-6, and TNF-α were detected by human ELISA kit (Meimian, Jiangsu, China) according to the manufacturer’s instructions.

### Proliferation assay

Cell proliferation assays for HaCaTs and HDFs were performed by Cell Counting Kit (CCK-8, YEASEN, Shanghai, China) following the manufacturing instructions. We seeded cells into 96-well culture plates with 1 × 10^3^ cells per well. CCK8 reagent was applied to the culture medium (100 μL per well) on days 1, 2, 3, 4, and 5. After incubation at 37 °C for 2 h, we measured the absorbance at 450 nm of each well by a microplate reader (Bio-Rad 680, Hercules, USA).

### Dual-luciferase reporter assay

The human HES1 promoter sequence was inserted into the firefly luciferase vector. Following UVB exposure, HDFs were incubated with FM, AMSC-EV, HUMSC-EV, TIMP1, GI254023X or VPA, as previously described. Then, HDFs were transfected with human-HES1-promoter-pGL3-Basic together with *Renilla* pRL-TK vector (Beyotime, Shanghai, China) using Lipofectamine 3000 (Invitrogen, Carlsbad, CA, USA) and cultured in 96 wells. Dual Glo Luciferase Reporter Gene Assay Kit (YEASEN, Shanghai, China) was applied to measure the luciferase activities at 48 h after transfection. Firefly luciferase activities were normalized to Renilla luciferase values.

### Statistical analysis

The data were presented as the mean ± standard deviation (SD). Statistical analysis was performed using GraphPad Prism 9.3.1 software. We analyzed the comparisons between groups using Student’s *t* test, one-way ANOVA followed by Tukey’s multiple comparisons test, and Dunnett’s multiple comparisons test. Differences were considered statistically significant when *P < 0.05, **P < 0.01, ***P < 0.001 and ****P < 0.0001.

## Supplementary information

Clean Supplementary Materials

## Data Availability

All data supporting this paper are present within the paper and/or the Supplementary Materials. The original datasets are also available from the corresponding author upon request. The raw data of RNA-seq presented in this study have been deposited in the NCBI Web site (https://www.ncbi.nlm.nih.gov), under the project number PRJNA1164396. The proteomics data have been deposited to the ProteomeXchange Consortium via the PRIDE partner repository with the dataset identifier PXD056371.
