# Supplementary material for: Human adipose and umbilical cord mesenchymal stem cell-derived extracellular vesicles mitigate photoaging via TIMP1/Notch1
Source: Signal Transduct Target Ther. 2024 Oct 30;9:294. doi: 10.1038/s41392-024-01993-z (PMC11522688; doi:10.1038/s41392-024-01993-z)
Supplement: Supplementary file 1 — Clean Supplementary Materials [file 41392_2024_1993_MOESM1_ESM.docx]

Supplementary Materials for

**Human adipose and umbilical cord mesenchymal stem cell-derived extracellular vesicles mitigate photoaging via TIMP1/Notch1**

Huan Zhang^1, #^, Xian Xiao^1, #^, Liping Wang^1^, Xianhao Shi^1^, Nan Fu^1^, Shihua Wang^1*^, Robert Chunhua Zhao^1, 2*^

#These authors contributed equally

Correspondence to: wangshihua@ibms.pumc.edu.cn or zhaochunhua@ibms.pumc.edu.cn.

**This PDF file includes:**

Figures. S1 to S12

Tables S1 to S2


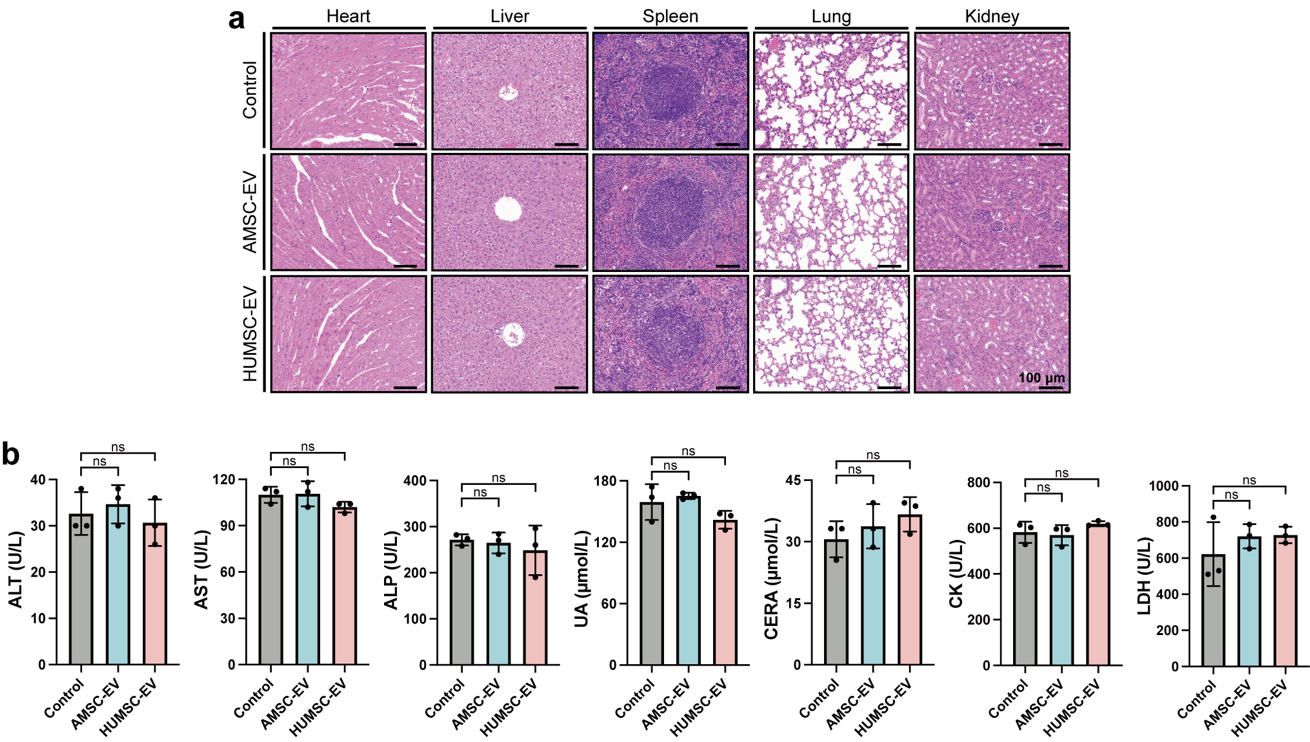
Figure. S1.

Figure S1: The safety assessment of AMSC-EV and HUMSC-EV in vivo.

(a), Histological morphologies of vital organs from mice at 72 hours after different treatments, n = 3.

(b), Blood biochemistry analysis of liver function indices, renal function indices and cardiac function indices, n = 3.


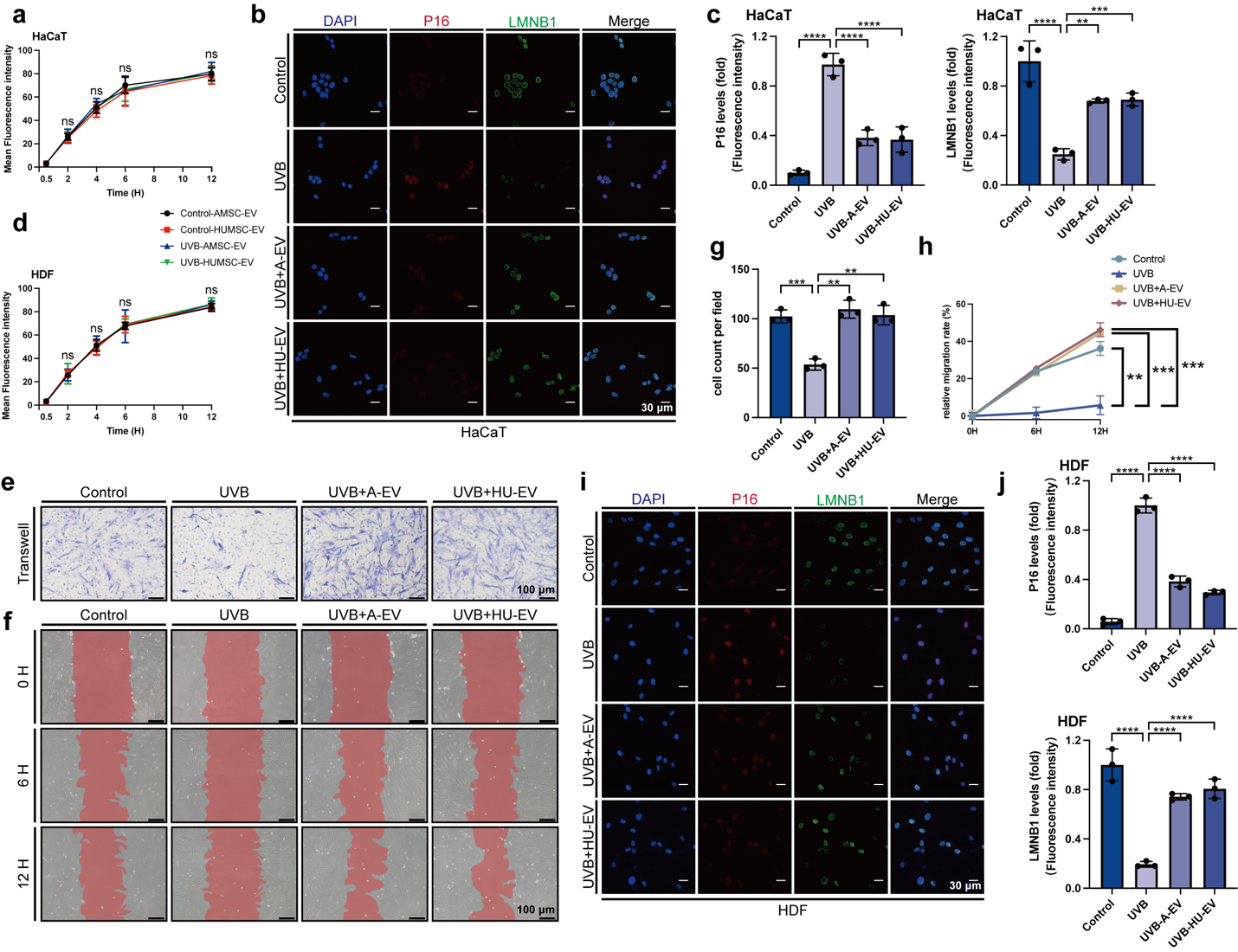
**Figure. S2.**

Figure S2: MSC-EV were uptaken by HDFs and HaCaTs and MSC-EV promoted HDFs migration.

(a), Fluorescence intensity analysis of AMSC-EV and HUMSC-EV uptake by HaCaTs pretreated with or without UVB, n = 3.

(b), Representative immunofluorescence staining images of P16 (red), LMNB1 (green) and DAPI of HaCaTs (scale bar, 30 μm).

(c), Fluorescence intensity of P16 and LMNB1 levels of HaCaTs. n = 3, **p < 0.01, ***p < 0.001, ****p < 0.0001.

(d), Fluorescence intensity analysis of AMSC-EV and HUMSC-EV uptake by HDFs pretreated with or without UVB, n = 3.

(e), Representative images of transwell assays of HDFs (scale bar, 100 μm).

(f), Representative images of the migration assay and the images were taken at the indicated times (scale bar, 100 μm).

(g), Quantitation of transwell assays of HDFs. n = 3, **p < 0.01, ***p < 0.001.

(h), Quantitation of migration assays of HDFs. n = 3, **p < 0.01, ***p < 0.001.

(i), Representative immunofluorescence staining images of P16 (red), LMNB1 (green) and DAPI of HDFs (scale bar, 30 μm).

(j), Fluorescence intensity of P16 and LMNB1 levels of HDFs. n = 3, ****p < 0.0001.


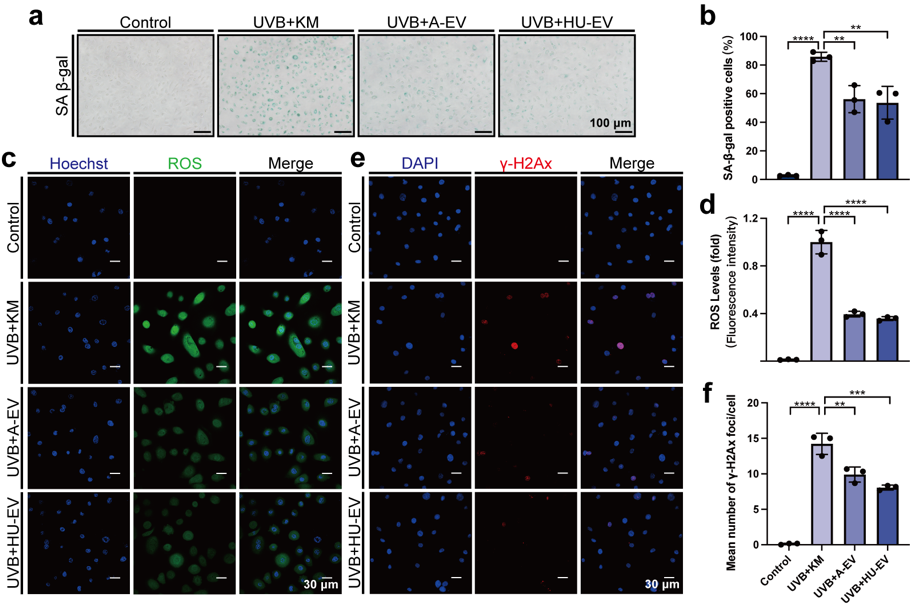
Figure. S3.

Figure S3: AMSC-EV and HUMSC-EV mitigated photoaging of HKCs in vitro.

(a), Representative images of SA-β-gal staining in HKCs (scale bar, 100 μm).

(b), Quantitation of SA-β-gal positive cells in HDFs. n = 3, **p < 0.01, ****p < 0.0001.

(c), Representative immunofluorescence staining images ROS (green) and DAPI (scale bar, 30 μm).

(d), Fluorescence intensity of ROS levels. n = 3, ****p < 0.0001.

(e), Representative immunofluorescence staining images of positive cells of γ-H2Ax (red) and DAPI (scale bar, 30 μm).

(f), Quantitation of mean number of γ-H2Ax foci/cell. n = 3, **p < 0.01, ****p < 0.0001.

**
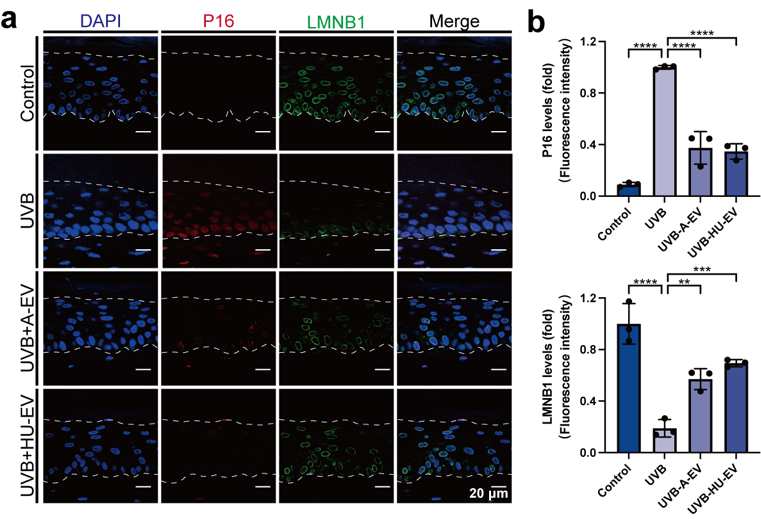
Figure. S4.**

Figure S4: AMSC-EV and HUMSC-EV mitigated photoaging in T-skin model.

(a), Representative immunofluorescence staining images of P16 (red), LMNB1 (green) and DAPI of T-Skin model (scale bar, 20 μm).

(b), Fluorescence intensity of P16 and LMNB1 levels of T-Skin model. n = 3, **p < 0.01, ***p < 0.001, ****p < 0.0001.

**
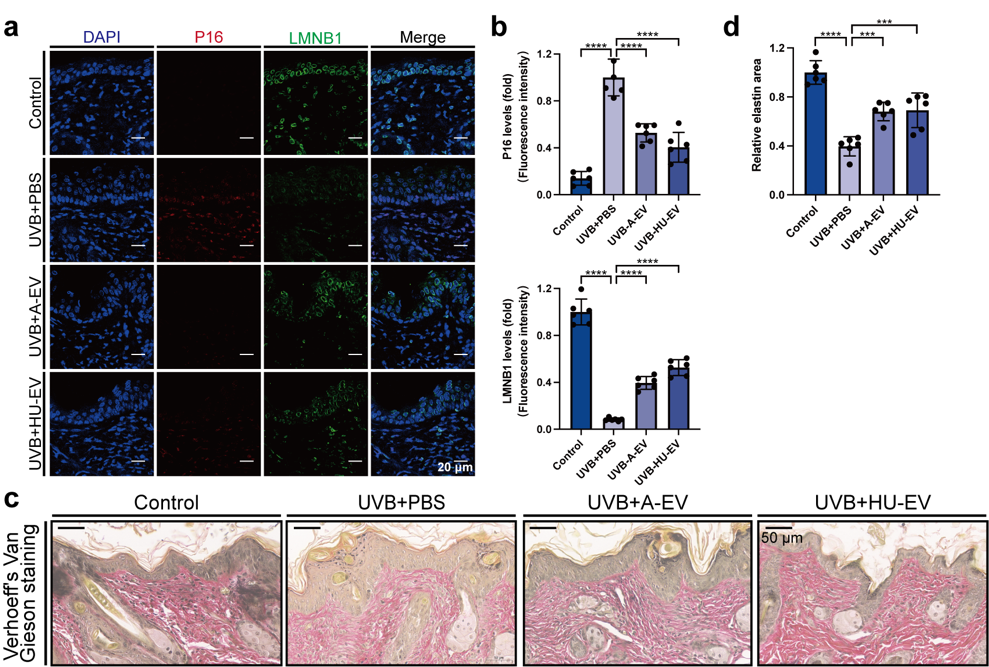
Figure. S5.**

Figure S5: Immunofluorescence and histological analysis of the dorsal skin in nude mice after UVB irradiation and EV treatment.

(a), Representative immunofluorescence staining images of P16 (red), LMNB1 (green) and DAPI of the dorsal skin in nude mice (scale bar, 30 μm).

(b), Fluorescence intensity of P16 and LMNB1 levels of the dorsal skin in nude mice. n = 6, ****p < 0.0001.

(c), Representative images of Verhoeff’s Van Gieson staining staining (scale bar, 50 μm).

(d), Quantitation of elastin area from Verhoeff’s Van Gieson staining staining statistics. n = 6, ***p < 0.001, ****p < 0.0001.


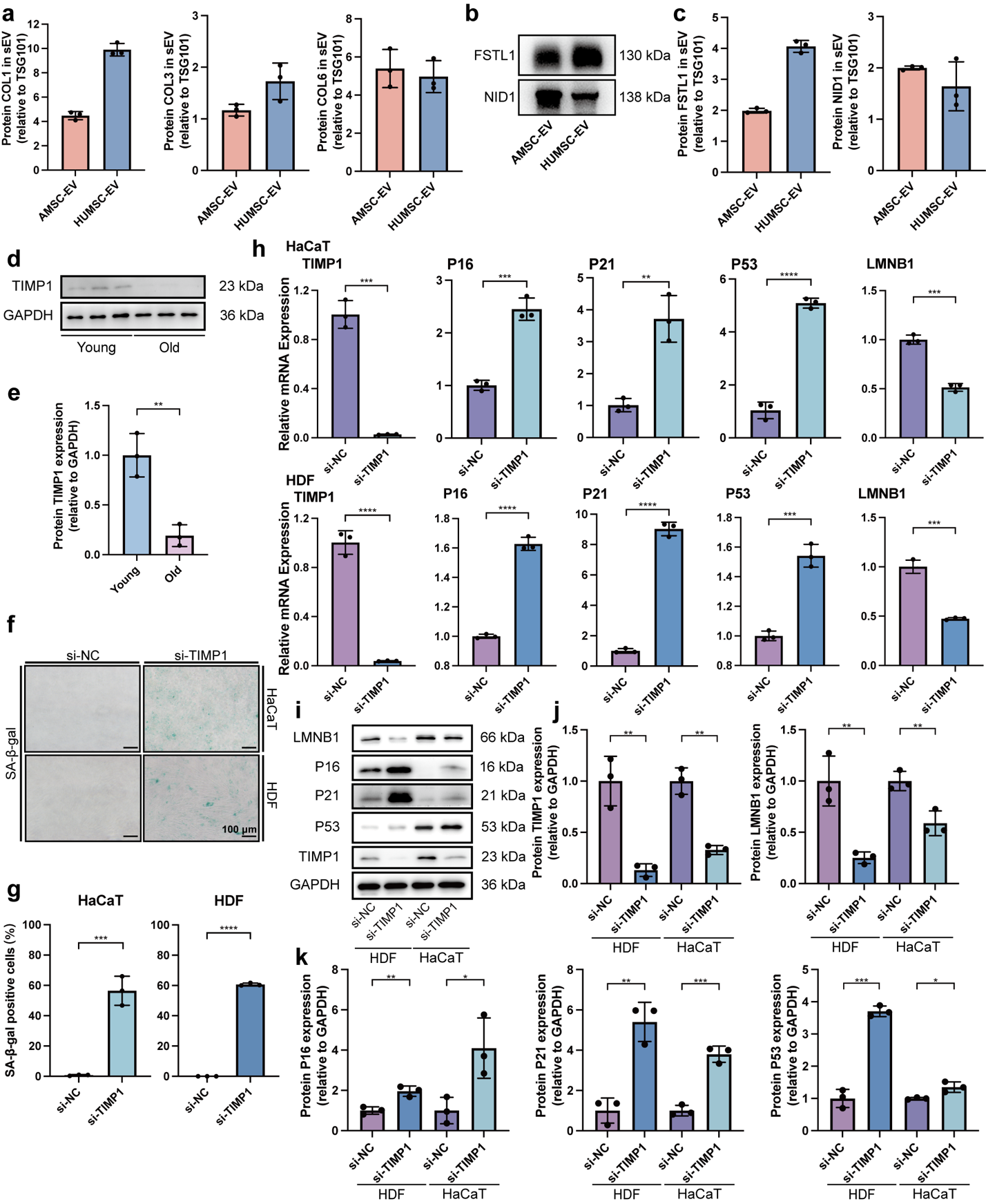
Figure. S6.

Figure S6: Silence of TIMP1 induces senescence in both HDFs and HaCaTs.

(a), Quantitation of COL1, COL3, and COL6 in AMSC-EV and HUMSC-EV was detected by Western blot. n = 3.

(b), Western blot analysis showing FSTL1 and NID1 expression in AMSC-EV and HUMSC-EV.

(c), Quantitation of FSTL1 and NID16 in AMSC-EV and HUMSC-EV was detected by Western blot. n = 3.

(d), Western blot analysis showing TIMP1 expression in both young and old mice. n = 3.

(e), Quantitative of TIMP1 in young and old mice by Western blot.

(f), Representative images of SA-β-gal staining in HaCaTs and HDFs (scale bar, 100 μm).

(g), Quantitation of SA-β-gal positive cells in HaCaTs and HDFs. n = 3, ***p < 0.001, ****p < 0.0001.

(h), Quantitation of TIMP1, P16, P21, P53, and LMNB1 in HaCaTs and HDFs by qRT-PCR. n = 3, **p < 0.01, ***p < 0.001, ****p < 0.0001.

(i), Western blot analysis showing LMNB1, P16, P21, P53 and TIMP1 expression in HDFs and HaCaTs.

(j), Quantitative of LMNB1 and TIMP1 in HDFs and HaCaTs by Western blot. n = 3, **p < 0.01.

(k), Quantitative of P16, P21, and P53 in HDFs and HaCaTs by Western blot. n = 3, *p < 0.05, **p < 0.01, ***p < 0.001.

**
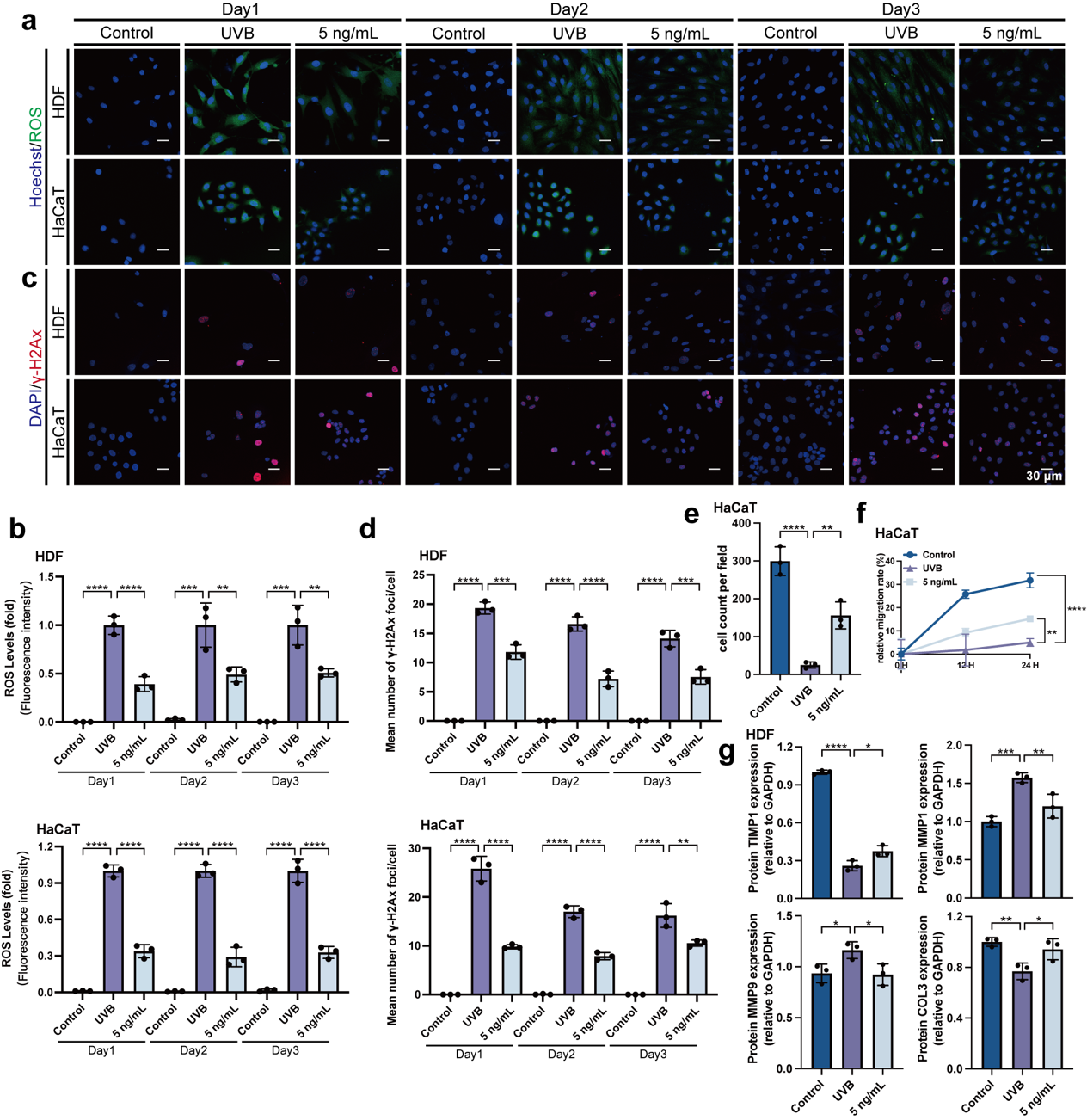
Figure. S7.**

Figure S7: MSC-EV rescues HDFs and HaCaTs photoaging by upregulating TIMP1.

(a), Representative immunofluorescence staining images of positive cells of ROS (green) and DAPI (scale bar, 35 μm).

(b), Quantitation of ROS-positive cells. n = 3, **p < 0.01, ***p < 0.001, ****p < 0.0001.

(c), Representative immunofluorescence staining images of positive cells of γ-H2Ax (red) and DAPI (scale bar, 20 μm).

(d), Quantitation of a mean number of γ-H2Ax foci/cell. n = 3, **p < 0.01, ***p < 0.001, ****p < 0.0001.

(e), Quantitation of transwell assays of HaCaTs. n = 3, **p < 0.01, ****p < 0.0001.

(f), Quantitation of migration assays of HaCaTs. n = 3, **p < 0.01, ****p < 0.0001.

(g), Quantitative of TIMP1, MMP1, MMP9, and TIMP1 in HDFs by Western blot. n = 3, *p < 0.05, **p < 0.01, ***p < 0.001, ****p < 0.0001.

**
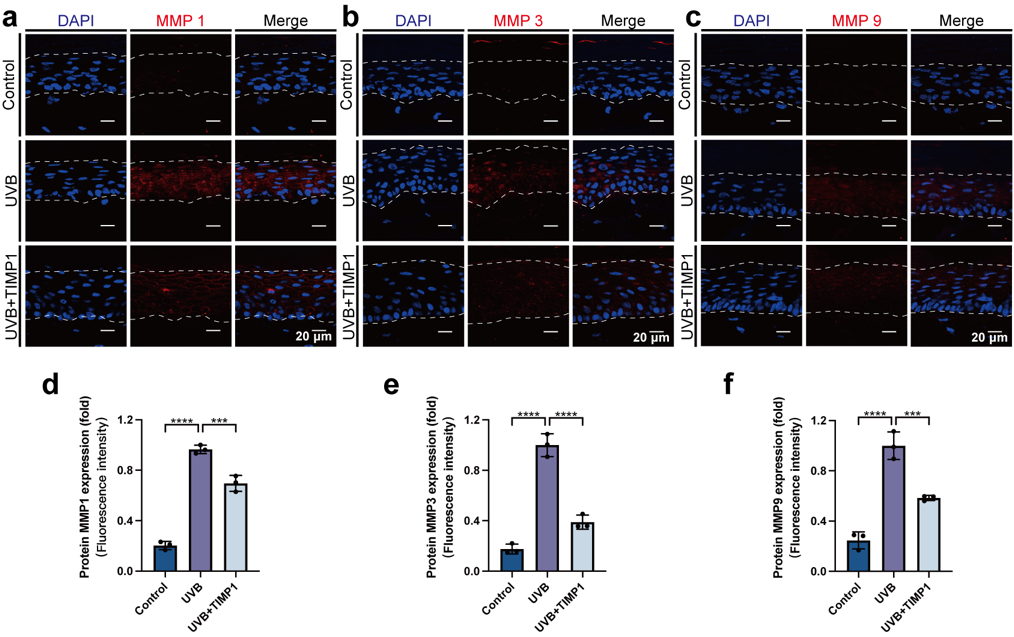
Figure. S8.**

Figure S8: TIMP1 reduced MMP1, MMP3, and MMP9 levels induced by UVB in T-skin model.

(a), Representative immunofluorescence staining images of MMP1 (red) and DAPI (scale bar, 20 μm).

(b), Representative immunofluorescence staining images of MMP3 (red) and DAPI (scale bar, 20 μm).

(c), Representative immunofluorescence staining images of MMP9 (red) and DAPI (scale bar, 20 μm).

(d), Fluorescence intensity of MMP1 levels. n = 3, ***p < 0.001, ****p < 0.0001.

(e), Fluorescence intensity of MMP3 levels. n = 3, ****p < 0.0001.

(f), Fluorescence intensity of MMP9 levels. n = 3, ***p < 0.001, ****p < 0.0001.


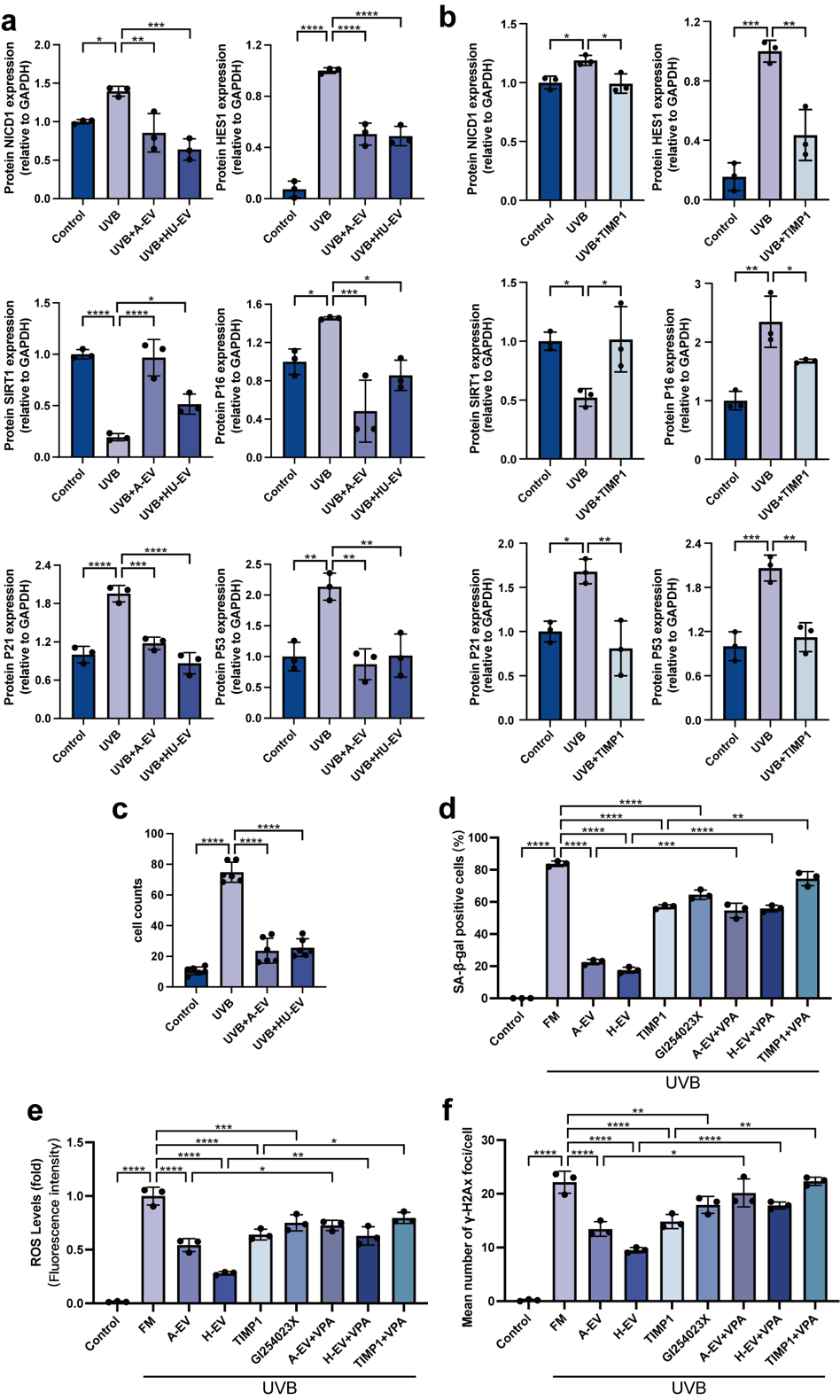
**Figure. S9.**

Figure S9: MSC-EV rescues HDFs photoaging by a downregulating NOTCH signaling pathway.

(a), Quantitation of NOTCH1, NICD1, HES1, SIRT1, P16, P21, and P53 by Western blot after EV treatment. n = 3, *p < 0.05, **p < 0.01, ***p < 0.001, ****p < 0.0001.

(b), Quantitation of NOTCH1, NICD1, HES1, SIRT1, P16, P21, and P53 by Western blot after TIMP1 treatment. n = 3, *p < 0.05, **p < 0.01, ***p < 0.001.

(c), Quantitation of positive cells of HES1 in nude mice dorsal skin injected PBS, AMSC-EV or HUMSC-EV. n = 6, ****p < 0.0001.

(d), Quantitation of SA-β-gal positive cells in HDFs. n = 3, **p < 0.01, ***p < 0.001, ****p < 0.0001.

(e), Fluorescence intensity of ROS levels. n = 3, *p < 0.05, **p < 0.01, ***p < 0.001, ****p < 0.0001.

(f), Quantitation of a mean number of γ-H2Ax foci/cell. n = 3, *p < 0.05, **p < 0.01, ****p < 0.0001.

**
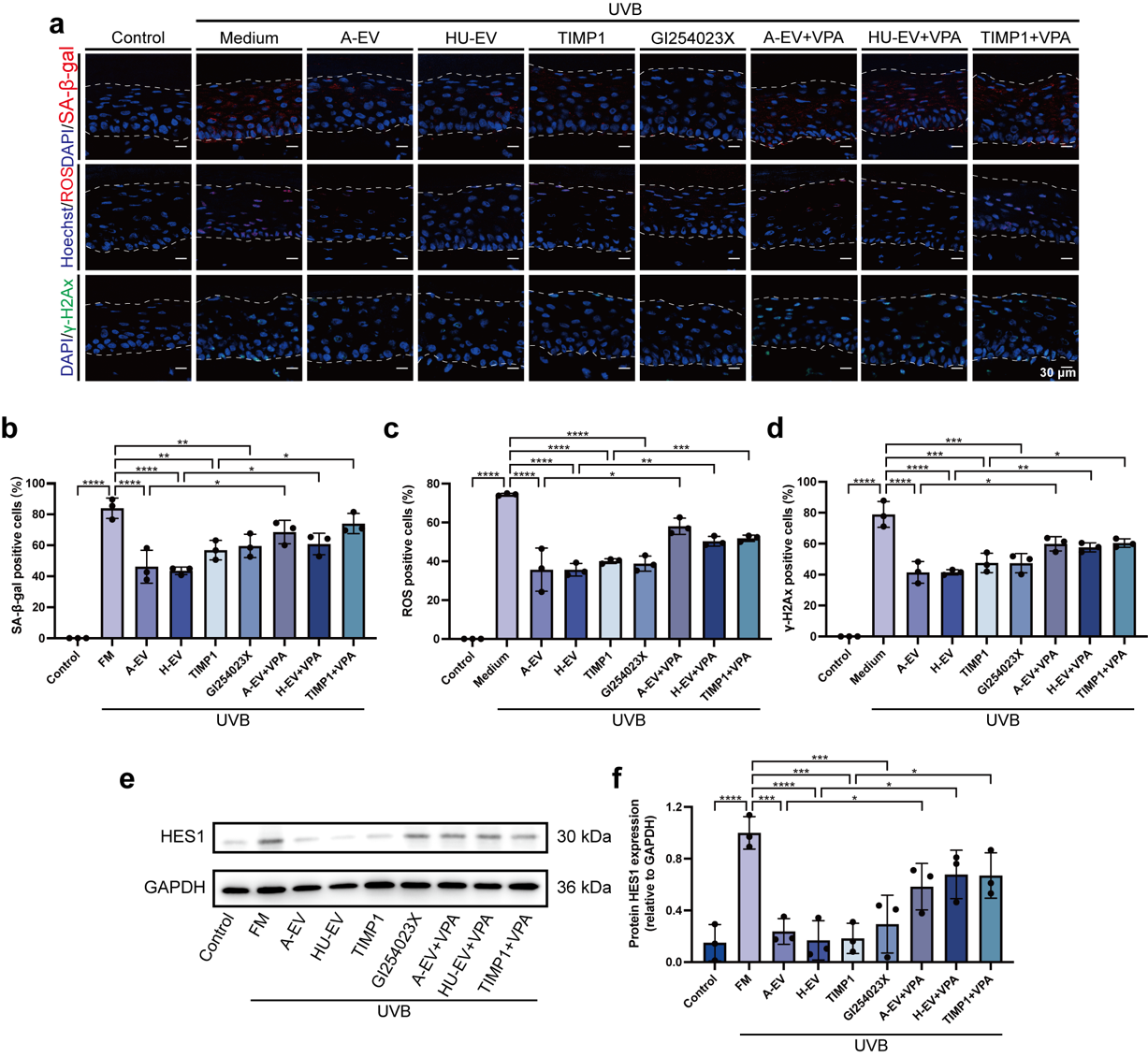
Figure. S10.**

Figure S10: MSC-EV rescues HDFs photoaging by a downregulating NOTCH signaling pathway in T-Skin model.

(a), Representative immunofluorescence staining images of SA-β-gal (red), ROS (red) and γ-H2Ax (green) (scale bar, 30 μm).

(b), Quantitation of SA-β-gal positive cells in T-Skin model. n = 3, *p < 0.05, **p < 0.01, ***p < 0.001, ****p < 0.0001.

(c), Quantitation of ROS positive cells in T-Skin model. n = 3, *p < 0.05, **p < 0.01, ***p < 0.001, ****p < 0.0001.

(d), Quantitation of γ-H2Ax positive cells in T-Skin model. n = 3, *p < 0.05, **p < 0.01, ***p < 0.001, ****p < 0.0001.

(e), Western blot analysis showing expression of HES1 and GAPDH after UVB, FM (culture medium), EV, TIMP1, GI254023X, and VPA treatment.

(f), Quantitation of HES1 by Western blot after UVB, FM (culture medium), EV, TIMP1, GI254023X, and VPA treatment. n = 3, *p < 0.05, ***p < 0.001, ****p < 0.0001.

**
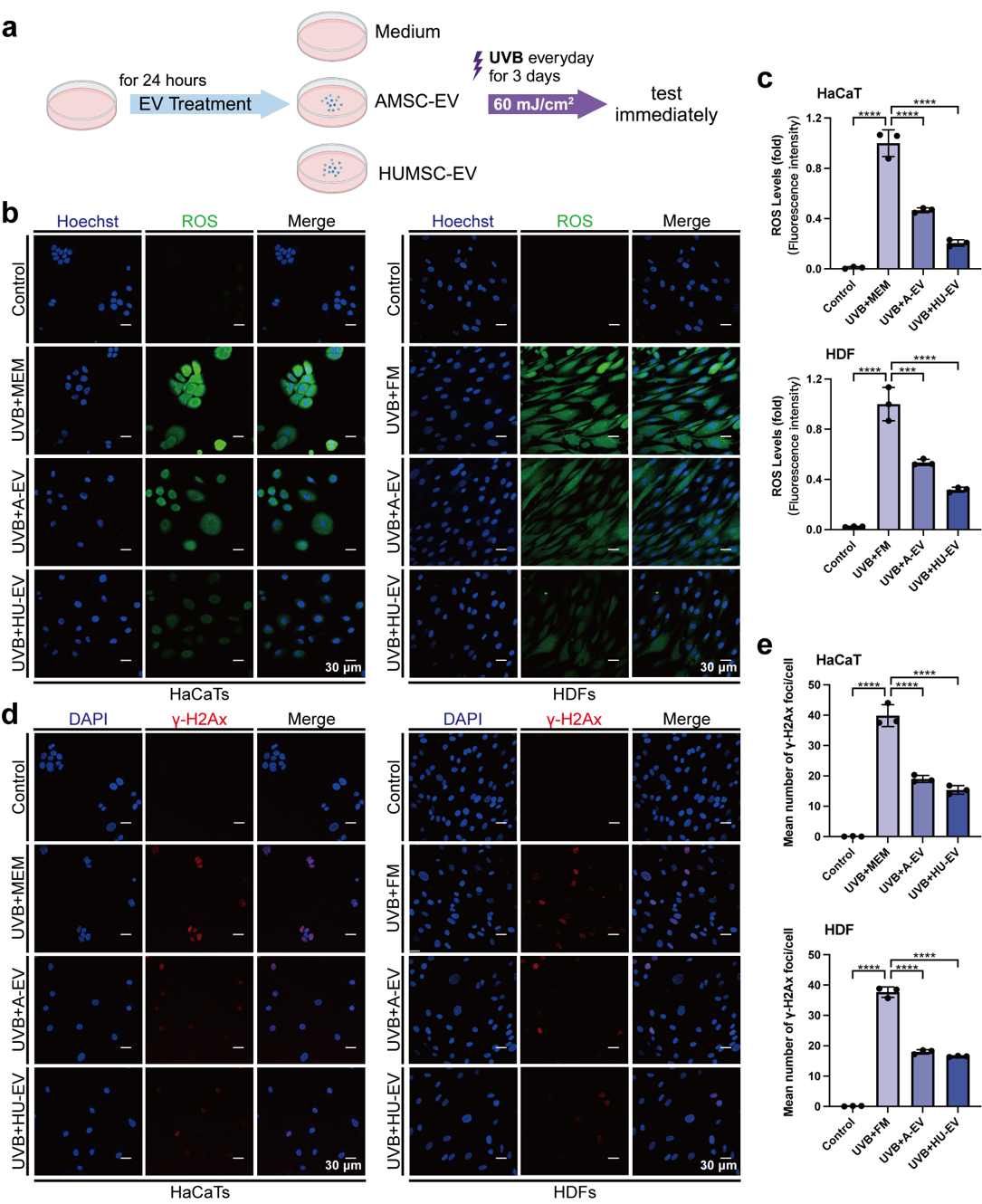
Figure. S11.**

Figure S11: Pre-treatment with AMSC-EV and HUMSC-EV before UVB exposure in HaCaTs and HDFs.

(a), Schematic representation of pre-treatment with AMSC-EV and HUMSC-EV before UVB exposure in HaCaTs and HDFs. (Created with BioRender.com)

(b), Representative immunofluorescence staining images ROS (green) and DAPI of HaCaTs and HDFs (scale bar, 30 μm).

(c), Fluorescence intensity of ROS levels of HaCaTs and HDFs. n = 3, ***p < 0.001, ****p < 0.0001.

(d), Representative immunofluorescence staining images of positive cells of γ-H2Ax (red) and DAPI of HaCaTs and HDFs (scale bar, 30 μm).

(e), Quantitation of mean number of γ-H2Ax foci/cell of HaCaTs and HDFs. n = 3, ****p < 0.0001.


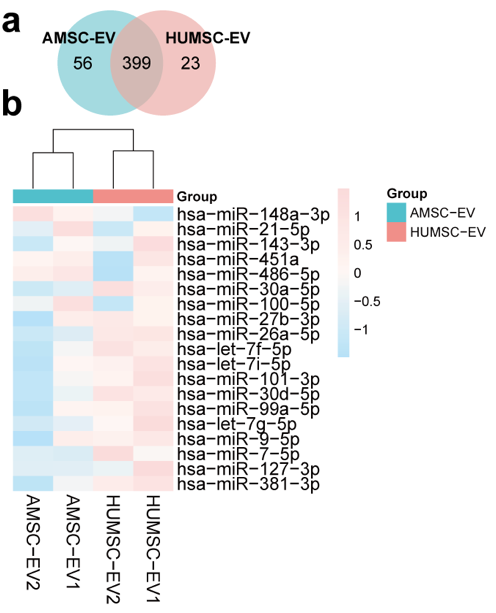
**Figure. S12.**

Figure S12: miRNA contents of AMSC-EV and HUMSC-EV.

(a), Venn diagram of the miRNA contents of AMSC-EV and HUMSC-EV.

(b), Heatmap of the common TOP 20 miRNA contents of AMSC-EV and HUMSC-EV.

Table S1. Sequences of primers and siRNA

| **Primer name** | **Sequences (5′-3′)** |
| --- | --- |
| hGAPDH-F | ACATCGCTCAGACACCATG |
| hGAPDH-R | TGTAGTTGAGGTCAATGAAG |
| hMMP1-F | ATGAAGCAGCCCAGATGTGGAG |
| hMMP1-R | TGGTCCACATCTGCTCTTGGCA |
| hMMP9-F | TGTACCGCTATGGTTACACTCG |
| hMMP9-R | GGCAGGGACAGTTGCTTCT |
| hTIMP1-F | CTTCTGCAATTCCGACCTCGT |
| hTIMP1-R | ACGCTGGTATAAGGTGGTCTG |
| hCOL3A1-F | GTT TTG CCC CGT ATT ATG GA |
| hCOL3A1-R | GGA AGT TCA GGA TTG CCG TA |
| hIL-1β-F | ATGATGGCTTATTACAGTGGCAA |
| hIL-1β-R | GTCGGAGATTCGTAGCTGGA |
| hIL-6-F | ACTCACCTCTTCAGAACGAATTG |
| hIL-6R | CCATCTTTGGAAGGTTCAGGTTG |
| hTNF-a-F | CAAAGTAGACCTGCCCAGAC |
| hTNF-a-R | GACCTCTCTCTAATCAGCCC |
| hHes1-F | TCAACACGACACCGGATAAAC |
| hHes1-R | GCCGCGAGCTATCTTTCTTCA |
| hHeyL-F | ATGAGTCCTGGGAGAGACCC |
| hHeyL-R | GCCAGTCAGTCATTGCTCCT |
| hDLL1-F | CTTCCCCTTCGGCTTCAC |
| hDLL1-R | GGGTTTTCTGTTGCGAGGT |
| hLFNG-F | GTCAGCGAGAACAAGGTGC |
| hLFNG-R | GATCCGCTCAGCCGTATTCAT |
| hTle1-F | CCAGTACCTCTCACGCCTCA |
| hTle1-F | GCCCACTCAGAGCACTAGAC |
| P53-F1 | CAGCACATGACGGAGGTTGT |
| P53-R1 | TCATCCAAATACTCCACACGC |
| P16-F1 | GATCCAGGTGGGTAGAAGGTC |
| P16-R1 | CCCCTGCAAACTTCGTCCT |
| P21-F1 | TGTCCGTCAGAACCCATGC |
| P21-R1 | AAAGTCGAAGTTCCATCGCTC |
| LMNB1-F | AAGCATGAAACGCGCTTGG |
| LMNB1-R | AGTTTGGCATGGTAAGTCTGC |

| Sequences of siRNA |  |
| --- | --- |
| Name | **Sequences** |
| si-TIMP1-F | GCAAUUCCGACCUCGUCAUTT |
| si-TIMP1-R | AUGACGAGGUCGGAAUUGCTT |

Table S2. Antibody

| **Antibody name** | **Purchasing companies** | **Cat No** |
| --- | --- | --- |
| CD63 | Proteintech | 25682-1-AP |
| CD9 | Abcam | ab92726 |
| TSG101 | Abcam | ab125011 |
| Alix | Cell Signaling technology | 92880 |
| Calnexin | Cell Signaling technology | 2433 |
| MMP1 | Proteintech | 10371-2-AP |
| MMP9 | Proteintech | 10375-2-AP |
| TIMP1 | Proteintech | 6644-1-AP |
| Collagen III | Abcam | ab7778 |
| GAPDH | Proteintech | 10494-1-AP |
| FSTL1 | Proteintech | 20182-1-AP |
| Entactin | Proteintech | 13766-1-AP |
| NOTCH1 | Proteintech | 20687-1-AP |
| P16-INK4A | Proteintech | 10883-1-AP |
| P21 | Proteintech | 10355-1-AP |
| P53 | Proteintech | 10442-1-AP |
| SIRT1 | Proteintech | 13161-1-AP |
| HES1 | Cell Signaling technology | 11988S |
| Lamin B1 | Proteintech | 12987-1-AP |
| beta galactosidase | Proteintech | 15518-1-AP |
| P16-INK4A | Abcam | ab241543 |
| Lamin B1 | Abcam | ab16048 |
